# Delayed recruiting of TPD52 to lipid droplets – evidence for a “second wave” of lipid droplet-associated proteins that respond to altered lipid storage induced by Brefeldin A treatment

**DOI:** 10.1038/s41598-019-46156-1

**Published:** 2019-07-05

**Authors:** Yuyan Chen, Sarah Frost, Matloob Khushi, Laurence C. Cantrill, Hong Yu, Jonathan W. Arthur, Robert K. Bright, Guy E. Groblewski, Jennifer A. Byrne

**Affiliations:** 1 Molecular Oncology Laboratory, Children’s Cancer Research Unit, Kids Research, The Children’s Hospital at Westmead, Westmead, NSW 2145 Australia; 2 The University of Sydney Discipline of Child and Adolescent Health, The Children’s Hospital at Westmead, Westmead, NSW 2145 Australia; 3 Bioinformatics Unit, Children’s Medical Research Institute, The University of Sydney, Westmead, NSW 2145 Australia; 4 The University of Sydney School of Information Technologies, Darlington, NSW 2008 Australia; 5 Kids Research Microscope Facility, The Children’s Hospital at Westmead, Westmead, NSW 2145 Australia; 6 Cell Imaging Facility, Westmead Institute for Medical Research, Westmead, NSW 2145 Australia; 7 Department of Immunology and Molecular Microbiology and TTUHSC Cancer Center, Texas Tech University Health Sciences Center, Lubbock, Texas 79430 USA; 8 Department of Nutritional Sciences, University of Wisconsin-Madison, Madison, Wisconsin 53706 USA

**Keywords:** Cancer metabolism, Golgi

## Abstract

*Tumor protein D52 (TPD52)* is amplified and overexpressed in breast and prostate cancers which are frequently characterised by dysregulated lipid storage and metabolism. TPD52 expression increases lipid storage in mouse 3T3 fibroblasts, and co-distributes with the Golgi marker GM130 and lipid droplets (LDs). We examined the effects of Brefeldin A (BFA), a fungal metabolite known to disrupt the Golgi structure, in TPD52-expressing 3T3 cells, and in human AU565 and HMC-1-8 breast cancer cells that endogenously express TPD52. Five-hour BFA treatment reduced median LD numbers, but increased LD sizes. TPD52 knockdown decreased both LD sizes and numbers, and blunted BFA’s effects on LD numbers. Following BFA treatment for 1–3 hours, TPD52 co-localised with the trans-Golgi network protein syntaxin 6, but after 5 hours BFA treatment, TPD52 showed increased co-localisation with LDs, which was disrupted by microtubule depolymerising agent nocodazole. BFA treatment also increased perilipin (PLIN) family protein PLIN3 but reduced PLIN2 detection at LDs in TPD52-expressing 3T3 cells, with PLIN3 recruitment to LDs preceding that of TPD52. An N-terminally deleted HA-TPD52 mutant (residues 40–184) almost exclusively targeted to LDs in both vehicle and BFA treated cells. In summary, delayed recruitment of TPD52 to LDs suggests that TPD52 participates in a temporal hierarchy of LD-associated proteins that responds to altered LD packaging requirements induced by BFA treatment.

## Introduction

Lipid droplets (LDs) are complex and dynamic cytoplasmic organelles that not only supply cellular lipids for energy metabolism, membrane synthesis, but also produce essential lipid-derived signalling molecules^1–3^. LDs have increasingly received attention due to their critical roles in metabolic diseases such as lipodystrophies, obesity and type 2 diabetes, and in infectious diseases including hepatitis C^4^.

LDs are uniquely composed of a hydrophobic core of neutral lipids (including triacylglycerols and cholesteryl esters) surrounded by a monolayer of amphipathic lipids and associated proteins^1–3,5^. The numbers and sizes of LDs may vary considerably depending on cell type, and may also vary between individual cells of the same type. Furthermore, changing the metabolic state of the cell can cause rapid changes in LD numbers and sizes^2^. Fatty acid uptake or *de novo* adipogenesis results in increased LD numbers and/or sizes, whereas LDs shrink during cell starvation^2^. A large number of studies indicate that LD formation is initiated in the endoplasmic reticulum (ER), with so-called initial LDs ranging from 300–600 nm in diameter^2,6–8^. A subset of initial LDs can become expanding LDs (∼µm in diameter) with a distinct protein composition including triglyceride (TG) synthesis enzymes (e.g. GPAT4, AGPAT3, DGAT2) that mediate their expansion^9–11^. Large LDs can also arise via fusion or coalescence of LDs through SNARE proteins^12^ or fat-specific protein 27 (FSP27/CIDEC)^13,14^.

LDs are characterised by numerous proteins associated with their surfaces that execute distinct functions, and these proteins are targeted to LDs via different mechanisms^6,8^. Proteins can be attached to the LD surface from the ER through hairpin helices (e.g. GPAT4, caveolins), from the cytoplasm via amphipathic helices [e.g. perilipin (PLIN) family proteins], using lipid anchors (e.g. small GTPase Rab18), or by binding to other LD proteins (e.g. hormone-sensitive lipase/HSL)^6,8^. Recently, Prevost *et al*. have demonstrated that amphipathic helices with large hydrophobic residues are capable of binding to LDs^15^. On the other hand, proteins can be removed through protein crowding when the LD surface area decreases dramatically during lipolysis, and then degraded by the ubiquitin/proteasome system (e.g. adipophilin/PLIN2)^16,17^, or re-localised to the ER (e.g. Ubxd8, AAM-B)^8,18^.

Genome-wide screening studies have linked the Golgi apparatus with LD biogenesis^19,20^. As a central hub for intracellular membrane traffic in the eukaryotic cell, the Golgi apparatus is compartmentalised into *cis*, *medial*, and *trans*-cisternae, as well as the trans-Golgi network (TGN). Studies with Brefeldin A (BFA), a fungal metabolite, have provided important insights into intracellular membrane traffic and Golgi functions. BFA inhibits guanine nucleotide exchange factors (GEF) which prevents the activation of the ADP-ribosylation factor (ARF) family of small GTPases, resulting in dissociation of cytosolic coat protein complexes including COPI, clathrin/AP-1, and GGAs (Golgi-localising, γ-adaptin ear homology domain, ARF-binding proteins) from the cis-Golgi, or the TGN membranes, respectively^21–26^. At the same time, BFA treatment induces extensive retrograde transport of *cis-*. *medial*, and *trans*-Golgi components to the ER, leading to the complete loss of Golgi structure^27–29^. Genome-wide screening has identified the ARF1/COPI machinery, which regulates the retrograde vesicular trafficking from Golgi to ER, as a regulator of lipid homeostasis^19,20^. Knockdown of components of the COPI complex resulted in dramatically increased lipid storage, partially due to defective coatomer-dependent protein delivery to LDs, including that of adipose triglyceride lipase (ATGL)^19^. It has further been shown that the ARF1/COPI machinery locates to LDs and enables ER-LD bridging to target TG-synthesis enzymes from ER to LD surfaces^10,11^. By characterising the phenotype of adipocyte-specific *Arfrp1* (*ARF-related protein 1*)-null mutant mice, it was also proposed that the trans-Golgi GTPase ARFRP1-regulated ARFRP1-ARL1 (ARF-like 1)-Rab-Golgin cascade is involved in LD fusion and lipolysis^30,31^.

Amplification and/or overexpression of *Tumor protein D52* (*TPD52*) has been frequently identified in breast and prostate cancer^32^, both of which are frequently characterised by lipogenic phenotypes^33^. Our group has identified that TPD52 increases lipid storage in cultured cells^34^. In TPD52-expressing 3T3 cells, TPD52 showed cytoplasmic staining with predominant localisation at the perinuclear region which co-distributed with the Golgi marker GM130^34^. A small proportion of TPD52 also co-localised with PLIN2-coated LDs under routine culture conditions, with this co-localisation being significantly increased upon oleic acid supplementation^34^. To better understand the possible roles that TPD52 may play at both the Golgi apparatus and LD, we exploited BFA to further study Golgi function and LD biology in cell lines that either exogenously or endogenously express TPD52.

## Results

### BFA treatment decreased LD numbers but increased LD sizes in D52-2-7 cells

**Figure 1:**
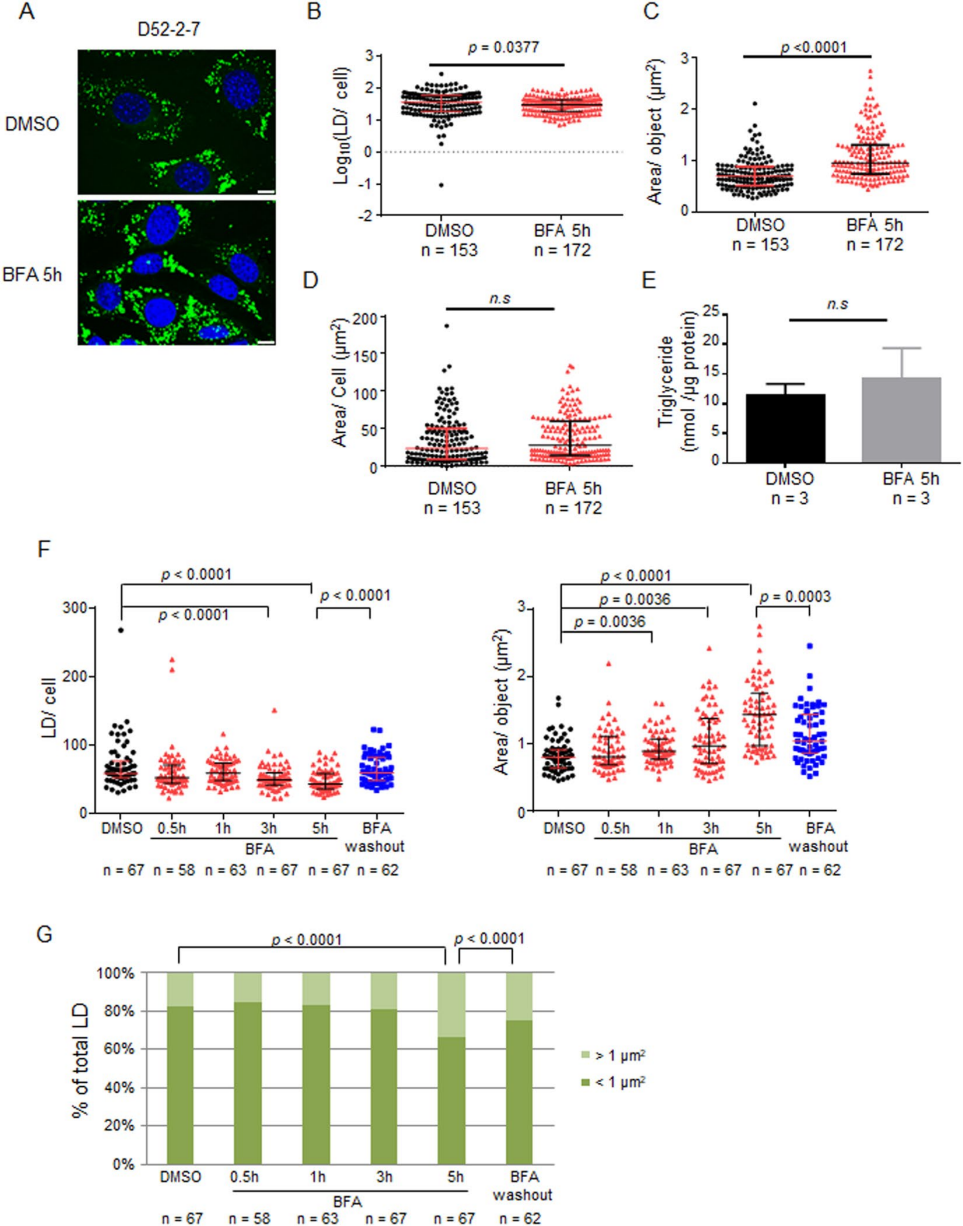
BFA treatment decreased LD numbers but increased LD sizes in D52-2-7 cells. (**A**) Representative images of LDs stained with BODIPY 493/503 (green) and nuclei stained with DAPI (blue) in D52-2-7 cells treated with vehicle (0.02% (v/v) DMSO), or 2 µg/ml (7.1 µM) BFA for 5 h. Scale bar = 10 μm. (**B–D**) Quantification of (**B**) LD numbers/cell (Log10 scale, Y axis), (**C**) LD areas (μm^2^)/object (Y axis), and (**D**) total LD areas/cell (Y axis) from the indicated numbers of images (below X axes) obtained from 6 independent experiments of D52-2-7 cells treated with vehicle (DMSO, black circles) or BFA (red triangles) for 5 h. Horizontal lines indicate median values, bounded by interquartile range. *P* values, Mann Whitney *u* test. (**E**) Triglyceride levels (Y axis, nmol/µg protein, mean values +/− s.e.m values from 3 independent experiments) measured in vehicle (black) or BFA-treated (grey) cells as describe above. n.s = not statistically significant, Student’s t-test. (**F**) Quantification of LD numbers/cell (Y axis, left), and LD areas (μm^2^)/object (Y axis, right) from the indicated numbers of images (below X axes) obtained from 3 independent experiments of D52-2-7 cells treated with DMSO vehicle for 5 h (black circles), or BFA for indicated time periods (red triangles), or following PBS washout after 5 h BFA treatment, and incubation at 37 °C for 1 h in complete media without BFA (BFA washout, blue squares). Horizontal lines indicate median values, bounded by interquartile ranges. *P* values, Mann Whitney *u* test. (**G**) Quantification of percentages (Y axis) of LDs with area >1 μm^2^ (light green) or ≤1 μm^2^ (dark green) in D52-2-7 cells after treatments described in (**F**) (X axis). *P* value, Pearson’s Chi-Squared test.

BFA inhibits the assembly of the COPI coat^28,35,36^, and may increase cellular lipid storage by regulating coatomer-dependent protein delivery^10,19,37^. We performed BODIPY 493/503 immunofluorescence analysis of LDs in BALB/c 3T3 stably TPD52-expressing D52-2-7 cells treated with 0.02% (v/v) DMSO as vehicle or 2 µg/ml (7.1 µM) BFA for 5 h. Quantification of LDs revealed that BFA treatment resulted in a significant ~15% decrease in LD numbers per cell, but a significant ~35% increase in LD sizes (Fig. 1A–C). This result may partly reflect increased LD clustering in BFA-treated cells, rendering the separation of individual objects more difficult using image analysis (Fig. 1A). Total LD areas per cell and triglyceride levels were not significantly altered in D52-2-7 cells after 5 h BFA treatment (Fig. 1D,E).

To further assess the kinetics of LD changes, we treated D52-2-7 cells with 2 µg/ml BFA for 0.5–5 h before fixation and immunofluorescence analyses. After 3 h BFA treatment, LD numbers/cell were significantly reduced, and further decreased after 5 h BFA treatment (Fig. 1F, left). However, LD sizes significantly increased after 1 h BFA treatment, and further increased after 3 h and 5 h BFA treatment (Fig. 1F, right). When we categorised LD sizes into >1 μm^2^ or ≤1 μm^2^, the percentage of LDs >1 μm^2^ nearly doubled following 5 h BFA treatment (Fig. 1G). After BFA washout and incubation in complete growth media for 1 h, both LD sizes and numbers partially recovered towards the levels measured in control cells (Fig. 1F,G).

### TPD52 knockdown in D52-2-7 cells decreased both LD numbers and sizes, and attenuated the effects of BFA

**Figure 2:**
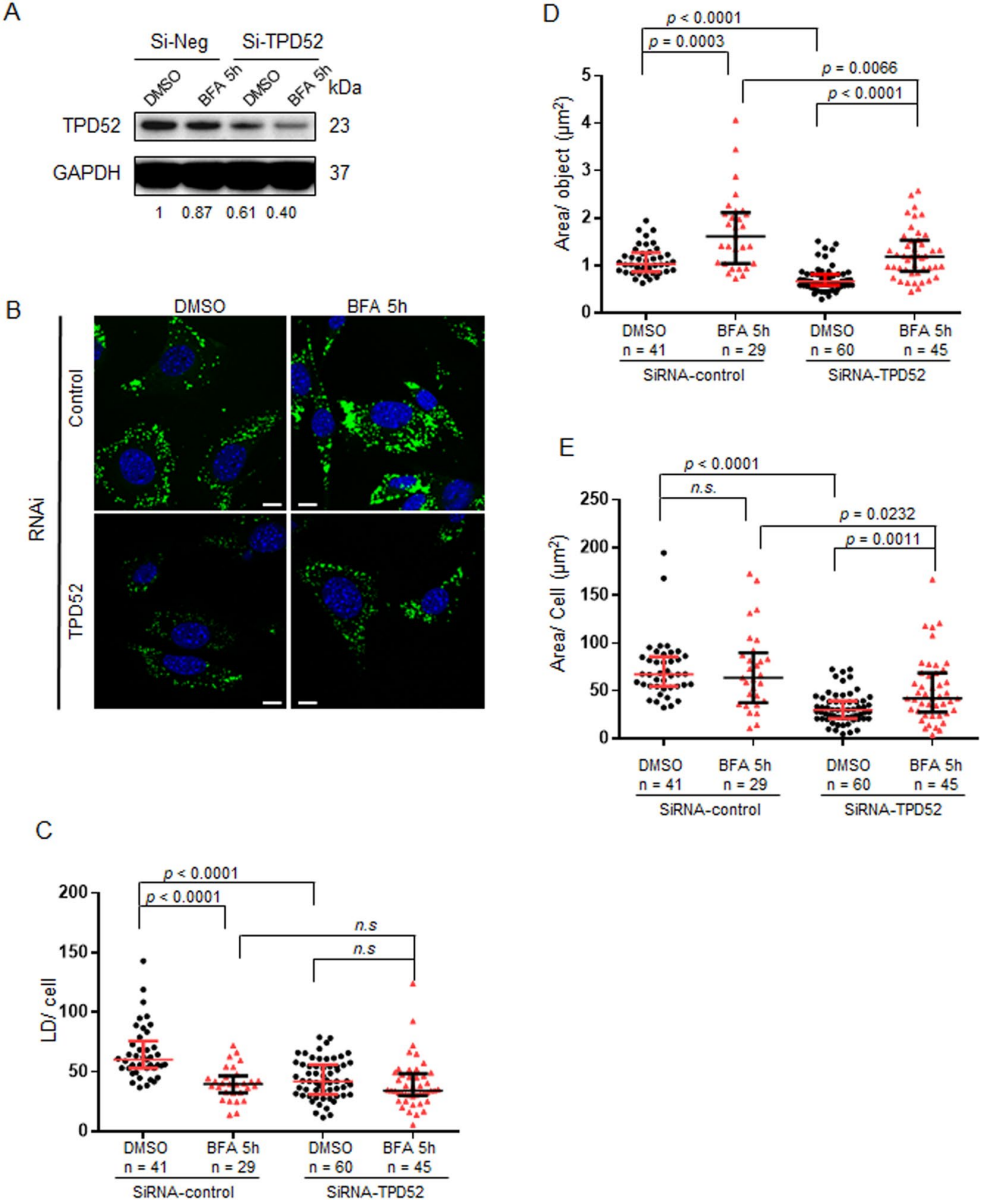
TPD52 knockdown decreased both LD numbers and sizes, and attenuated the effects of BFA on LD numbers in D52-2-7 cells. (**A**) Western blot analyses of D52-2-7 cells transfected with non-targeting siRNA (si-Neg) or *TPD52*-siRNA (si-TPD52) for 72 h and then treated with either vehicle (DMSO) or 2 µg/ml BFA for 5 h. Total protein extracts were subjected to Western blot analyses. GAPDH served as a loading control. Proteins are shown at left; molecular weights (kDa) are shown at right. Protein levels were quantified using Image J and normalised TPD52/GAPDH ratios (as described in the Methods) are indicated below the blots. See unprocessed Western blot images in Supplementary Fig. 11. (**B**) D52-2-7 cells were treated as described in (**A**), and then subjected to immunofluorescence analysis with LDs stained with BODIPY (green) and nuclei stained with DAPI (blue). Images shown are representative of those obtained in 3 independent experiments. Scale bar = 10 μm. (**C–E**) Quantification of (**C**) LD numbers/cell (Y axis), (**D**) LD areas (μm^2^)/object (Y axis), (**E**) LD areas (μm^2^)/cell in non-targeting siRNA-transfected, or *TPD52*-siRNA-transfected D52-2-7 cells following the indicated treatments (X axis) carried out as described in (**A**). Numbers of images quantified from vehicle-treated (DMSO, black circles), or BFA-treated (red triangles) cells obtained from 3 independent experiments are indicated below the X axes. Horizontal lines indicate median values, bounded by interquartile ranges. *P* values, Mann Whitney *u* test. n.s, not significant.

To investigate TPD52’s involvement in the effects of BFA, D52-2-7 cells were treated with a previously described *TPD52*-siRNA^38,39^, or non-targeting siRNA for 72 h, followed by DMSO or BFA treatment for 5 h. Western blot analysis confirmed reduced TPD52 levels in D52-2-7 cells treated with *TPD52*-siRNA, while BFA had no obvious impact on TPD52 levels (Fig. 2A). In non-targeting siRNA-treated cells, 5 h BFA treatment significantly decreased LD numbers/cell (Fig. 2B,C) but increased LD sizes (Fig. 2B,D), with no statistical differences in total LD areas/cell (Fig. 2E), which was consistent with the results shown in Fig. 1(B–D). In agreement with TPD52 expression increasing lipid storage^34^, TPD52 knockdown in vehicle-treated cells significantly decreased median LD numbers/cell (Fig. 2B,C), LD sizes (Fig. 2B,D), and total LD areas/cell (Fig. 2E), compared with non-targeting siRNA-transfected cells. In cells transfected with *TPD52*-siRNA, 5 h BFA treatment significantly increased LD sizes and total LD areas/cell compared with vehicle-treated cells (Fig. 2B,D,E), but did not significantly alter LD numbers/cell (Fig. 2B,C). In summary, TPD52 knockdown reduced LD numbers/cell, LD sizes, and total LD areas/cell in D52-2-7 cells, yet blunted BFA’s capacity to reduce LD numbers/cell.

### TPD52 sub-cellular redistribution post-BFA treatment

**Figure 3:**
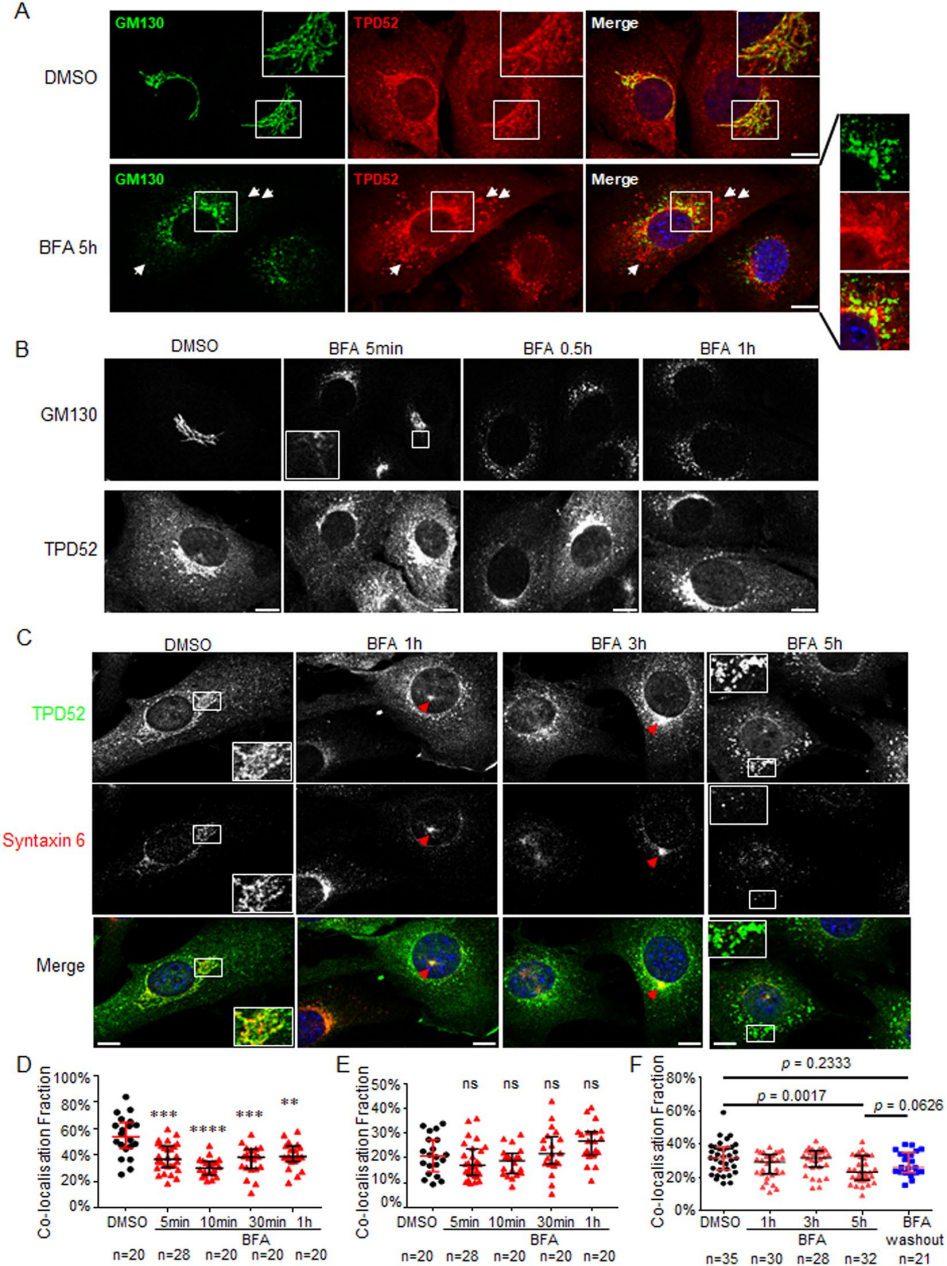
Redistribution of TPD52, GM130, and syntaxin 6 post-BFA treatment in D52-2-7 cells. (**A**) Indirect immunofluorescence analyses of D52-2-7 cells treated with vehicle control (DMSO) or 2 µg/ml BFA for 5 h, co-stained with GM130 (green) and TPD52 (red) with merged images. Enlarged images of white boxed regions indicate partial co-localisation of GM130 and TPD52 in vehicle-treated cells (DMSO), whereas co-localisation became more limited after BFA treatment. The ring structures indicated by white arrows in BFA-treated D52-2-7 cells were largely separate from the GM130-stained region. (**B**) Immunofluorescence analyses of D52-2-7 cells treated with vehicle (DMSO), or 2 µg/ml BFA for the indicated time periods, co-stained with GM130 and TPD52. The boxed region indicates the extended tabular network. (**C**) Immunofluorescence analyses of D52-2-7 cells treated with vehicle for 5 h (DMSO), or 2 µg/ml BFA for the indicated time periods, co-stained with syntaxin 6 (red) and TPD52 (green) with merged images. Enlarged images of white boxed regions indicate co-localisation between syntaxin 6 (red) and TPD52 (green) in DMSO-treated cells, contrasting with the comparatively limited syntaxin 6 and TPD52 co-localisation after 5 h BFA treatment. Red arrows indicate the co-staining of syntaxin 6 and TPD52 at the microtubule organising centre region after 1 h and 3 h BFA treatment. Images are representative of those obtained in 3 independent experiments. Scale bar = 10 μm. (**D**) Quantification of the fraction of GM130 co-localised with TPD52 using a custom-written MATLAB code as described in the Methods and supplementary information. Manders’ co-localisation coefficients (Y axis) from the indicated numbers of images (below the X axis) were obtained from D52-2-7 cells treated with DMSO vehicle for 1 h (black circles), or BFA for indicated time periods (red triangles). Horizontal lines indicate median values, bounded by interquartile ranges. ***p < 0.001 (DMSO vs BFA 5 min, DMSO vs BFA 30 min); ****p < 0.0001 (DMSO vs BFA 10 min); **p < 0.01 (DMSO vs BFA 1 h); Mann Whitney *u* test. (**E**) Quantification of the fraction of TPD52 co-localised with GM130 as described in (**D**). No significant differences (*ns*) were detected at each time point when compared with DMSO treatment; Mann Whitney *u* test. (**F**) Quantification of co-localisation fractions between syntaxin 6 and TPD52 using Manders’ co-localisation coefficients (Y axis) from the indicated numbers of images (below the X axis) from D52-2-7 cells treated with DMSO vehicle for 5 h (black circles), or BFA for indicated time periods (red triangles), or BFA washout as described in Fig. 1 (blue squares). Horizontal lines indicate median values, bounded by interquartile ranges. *P* values, Mann Whitney *u* test.

The most striking effects of BFA are the breakdown of the Golgi apparatus and rapid redistribution of Golgi proteins into the ER^27–29^. Our previous results have shown that in TPD52-expressing 3T3 cells, TPD52 co-localised with Golgi (GM130), but not with an ER marker^34^. We therefore compared the distributions of GM130 and TPD52 after BFA treatment. In vehicle-treated D52-2-7 cells, the Golgi apparatus stained with the cis-Golgi marker GM130 showed distinct perinuclear cisternae structures, with a proportion of TPD52 co-distributed at the same area (Fig. 3A, DMSO). Following 5 h BFA treatment, GM130 showed dispersed punctate staining, similar to the previously described redistribution of GM130 into the ER-Golgi intermediate compartment (ERGIC)^40^ (Fig. 3A, BFA 5 h). However, TPD52 staining in BFA-treated cells detected many ring structures in the cytoplasm, which were largely separate from the GM130-stained area (Fig. 3A, BFA 5 h). Consistent with the reported reversible effects of BFA on the Golgi apparatus^41–43^, D52-2-7 cells partially recovered the perinuclear co-distribution of GM130 and TPD52 at 1 h after BFA removal (Supplementary Fig. 1A).

The Golgi apparatus loses its compact perinuclear structure within minutes of BFA addition^42,43^. We sought to determine the dynamics of GM130 and TPD52 redistribution following BFA treatment in D52-2-7 cells. As expected, the Golgi showed evidence of an extended tubular network after 5 min of BFA exposure (Fig. 3B, GM130, inset), which is a hallmark of BFA’s morphological effects^27^. After 0.5 h BFA treatment, GM130 showed dispersed punctate staining (Fig. 3B, GM130). Surprisingly, while TPD52 co-distributed with GM130 at the juxta-nuclear region in DMSO-treated control cells (Fig. 3B), TPD52 retained perinuclear staining between 5 min to 1 h after BFA treatment when GM130 was dispersed (Fig. 3B). Quantification of the associated co-localisation fractions revealed that the fraction of GM130 co-localised with TPD52 was significantly reduced after 5 min–1 h BFA treatment (Fig. 3D), whereas the fraction of TPD52 that co-localised with GM130 was not similarly altered (Fig. 3E).

### TPD52 is localised at the trans-Golgi network (TGN)

The perinuclear staining of TPD52 in D52-2-7 cells treated with BFA (Fig. 3B, TPD52) was reminiscent of BFA-induced redistribution of TGN markers, which do not redistribute with markers of the *cis*/*medial*/*trans* Golgi into the ER during BFA treatment^29,44,45^. The TGN has a more tubular-vesicular structure than Golgi cisternae, in keeping with its function as a sorting site for proteins destined for different locations^46^. BFA treatment has been reported to induce the collapse of the TGN upon the microtubule-organising centre (MTOC)^29,44^. We therefore performed immunofluorescence analysis using syntaxin 6 as a TGN marker^47,48^.

Syntaxin 6 staining was enriched in the perinuclear region and showed partial co-localisation with GM130 under steady-state conditions in 3T3 parental, vector-transfected, or TPD52-expressing D52-2-7 cells (Supplementary Fig. 1C). TPD52 co-localised with syntaxin 6 at the perinuclear region in vehicle-treated D52-2-7 cells (Fig. 3C). After 1–3 h of BFA treatment, syntaxin 6 staining showed a tighter perinuclear or concentrated spherical distribution (Fig. 3C), reflecting typical TGN morphological changes induced by BFA^29,44,47,48^. Interestingly, a proportion of TPD52 clearly co-localised with syntaxin 6 at these regions (Fig. 3C). However, after 5 h BFA treatment, syntaxin 6 staining was detected as small, apparently vesicular structures distributed throughout the cytoplasm, whereas TPD52 staining detected many ring structures in the cytoplasm (Fig. 3C). Quantification confirmed that the median co-localisation fraction between syntaxin 6 and TPD52 significantly decreased after 5 h BFA treatment (Fig. 3F). At 1 h after BFA removal, D52-2-7 cells also partially recovered the co-distribution of syntaxin 6 and TPD52 (Fig. 3F and Supplementary Fig. 1B).

### Recruitment of TPD52 to LDs in BFA-treated D52-2-7 cells

**Figure 4:**
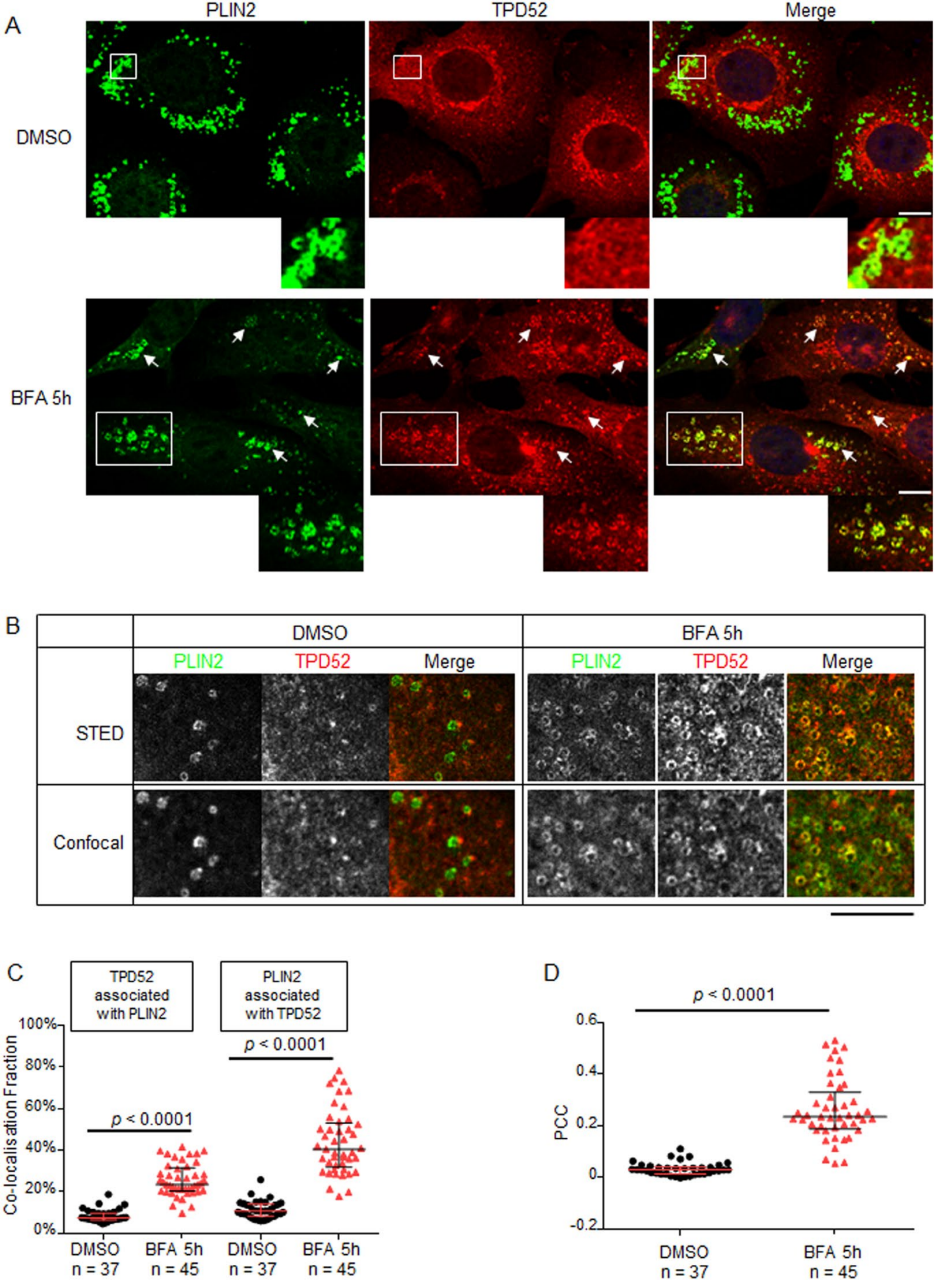
Significantly increased TPD52 detection at PLIN2-positive ring structures post-BFA treatment in D52-2-7 cells. (**A**) Immunofluorescence analyses of D52-2-7 cells treated with vehicle (DMSO) or 2 µg/ml BFA for 5 h, stained with PLIN2 (green) and TPD52 (red). White arrows indicate PLIN2- and TPD52-positive ring structures in BFA-treated cells. Enlarged images of white boxed regions indicate co-localisation between PLIN2 and TPD52 in vehicle-treated cells (DMSO), which became more prominent after BFA treatment. Images shown are representative of those obtained in 3 independent experiments. Scale bar = 10 μm. (**B**) STED imaging (top panel) and corresponding confocal images (bottom panel) of D52-2–7 cells treated as described in (**A**), stained with PLIN2 (pseudo-coloured green) and TPD52 (pseudo-coloured red) with merged images. Images are representative of those obtained from 2 independent experiments which were captured using Leica TCS SP8 STED 3X microsystem with a 100x objective lens. Scale bar = 8 μm. Quantification of (**C**) co-localisation fractions using Manders’ co-localisation coefficients (Y axis) and (**D**) Pearson’s correlation coefficients (PCC, Y axis) between PLIN2 and TPD52 from the indicated numbers of images (below X axes) obtained from 3 independent experiments where cells were treated with either vehicle (DMSO, black circles) or BFA (red triangles) as described in (**A**). Horizontal lines indicate median values, bounded by interquartile ranges. *P* values, Mann Whitney *u* test.

The finding of TPD52-positive ring structures induced by 5 h BFA treatment (Fig. 3A,C) led us to compare TPD52 and PLIN2 staining in D52-2-7 cells. Although there was limited detection of TPD52 at PLIN2-positive ring structures in control cells (Fig. 4A, DMSO), TPD52-stained ring structures were mostly co-stained with PLIN2 in cells treated with BFA for 5 h (Fig. 4A, BFA 5 h). The increased co-distribution of PLIN2 and TPD52 at LDs upon 5 h BFA treatment was further confirmed using super resolution STED microscopy (Fig. 4B). Quantification of the extent of co-localisation between PLIN2 and TPD52 revealed a significant increase following 5 h BFA treatment (Fig. 4C,D). We further analysed TPD52 detection at LDs post 5 h BFA-treatment using BODIPY staining (Supplementary Fig. 2). Quantification of the extent of overlap between BODIPY-stained LDs and TPD52 (Supplementary Fig. 2A) also revealed a significant increase in the extent of co-localisation (Supplementary Fig. 2B,C).

### BFA treatment reversibly promoted TPD52 detection at LDs in the breast cancer cell lines AU565 and HMC-1-8

**Figure 5:**
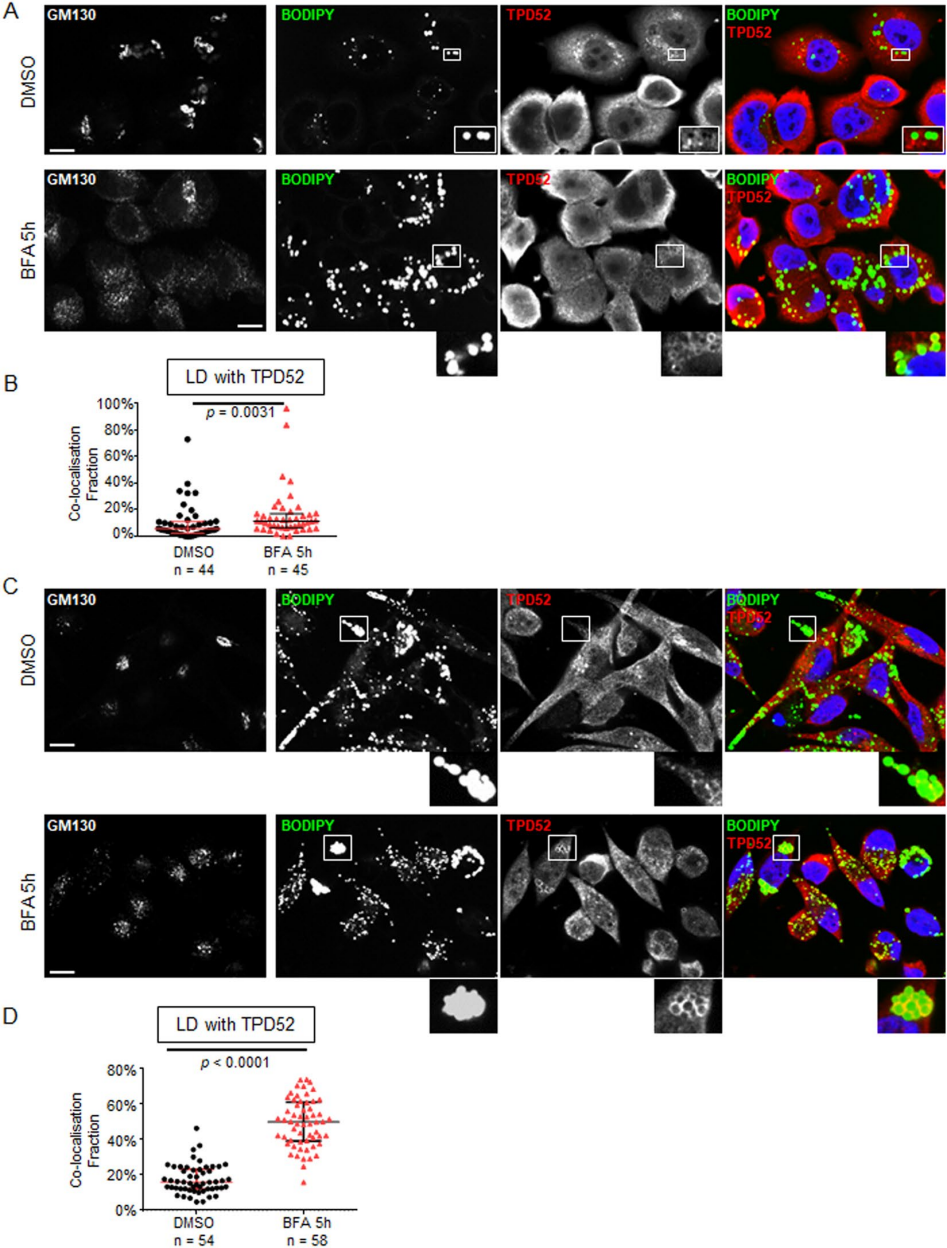
BFA treatment promoted TPD52 detection at LDs in AU565 and HMC-1-8 breast cancer cells. Immunofluorescence analyses of (**A**) AU565 and (**C**) HMC-1-8 cells following vehicle (DMSO) or 2 µg/ml BFA treatment for 5 h, stained with GM130, and BODIPY (green), TPD52 (red), and DAPI (blue), as shown in merged images at the right. Enlarged images of white boxed regions indicate co-localisation of TPD52 and BODIPY-stained LDs. Images are representative of those obtained in 3 independent experiments. Scale bar = 10 μm. (**B**,**D**) Quantification of co-localisation fractions (Manders’ co-localisation coefficients, Y axes) between BODIPY-stained LDs and TPD52 from the indicated numbers of images (below X axes) obtained from 3 independent experiments where AU565 (**B**) or HMC-1-8 (**D**) cells were treated with vehicle (DMSO, black circles) or BFA (BFA 5 h, red triangles). Horizontal lines indicate median values, bounded by interquartile ranges. *P* values, Mann Whitney *u* test.

To clarify whether the effects of BFA on TPD52 protein redistribution were limited to stably-transfected 3T3 cells, we examined the breast carcinoma cell lines AU565 and HMC-1-8^49^. The AU565 cell line, established from the same patient as the SK-BR-3 cell line, shows both *ERBB2* and *TPD52* amplification^50,51^, and higher ERBB2 and TPD52 protein levels than HMC-1-8 cells (Supplementary Fig. 3E). Immunofluorescence analyses revealed more prominent BODIPY 493/503 staining in HMC-1-8 cells under routine culture conditions compared with AU565 cells (Fig. 5A, DMSO and Fig. 5C, DMSO). Under the same conditions, TPD52 was detected as cytoplasmic staining which partially co-localised with GM130 (Supplementary Fig. 3A), but also co-localised with BODIPY-stained LDs in some AU565 and HMC-1-8 cells (Fig. 5A, DMSO and Fig. 5C, DMSO). After 2 µg/ml BFA treatment for 5 h, TPD52 showed markedly increased detection at LDs in both cell lines (Fig. 5A, BFA and 5 C, BFA). The median fraction of LDs co-localising with TPD52 increased nearly 2-fold in AU565 cells (Fig. 5B), and over 3-fold in HMC-1-8 cells (Fig. 5D). BFA treatment for 5 h also significantly increased LD sizes in both cell lines but only significantly decreased LD numbers per cell in HMC-1-8 cells (Supplementary Fig 3B,C). However, 5 h BFA treatment did not significantly alter triglyceride levels in either cell line (Supplementary Fig. 3D).

We also examined the PLIN proteins PLIN2 and PLIN3 in HMC-1-8 and AU565 cells. PLIN2 was weakly detected in both cell lines by Western blot analysis (Supplementary Fig. 3E), which was also confirmed by immunofluorescence analysis (data not shown). In contrast, PLIN3 was more strongly detected in HMC-1-8 cells than in AU565 cells (Supplementary Fig. 3E), so further experiments were conducted with HMC-1-8 cells. After 5 h BFA treatment, TPD52 detection significantly increased at LDs in HMC-1-8 cells (Supplementary Fig. 4A,B), whereas PLIN3 detection at LDs showed no significant change (Supplementary Fig. 4A,C). A partial recovery of TPD52 distribution was observed following BFA washout and 1 h recovery (Supplementary Fig. 4A,B).

### Recruitment of PLIN3 to LDs and loss of PLIN2 from LDs in BFA-treated D52-2-7 cells

**Figure 6:**
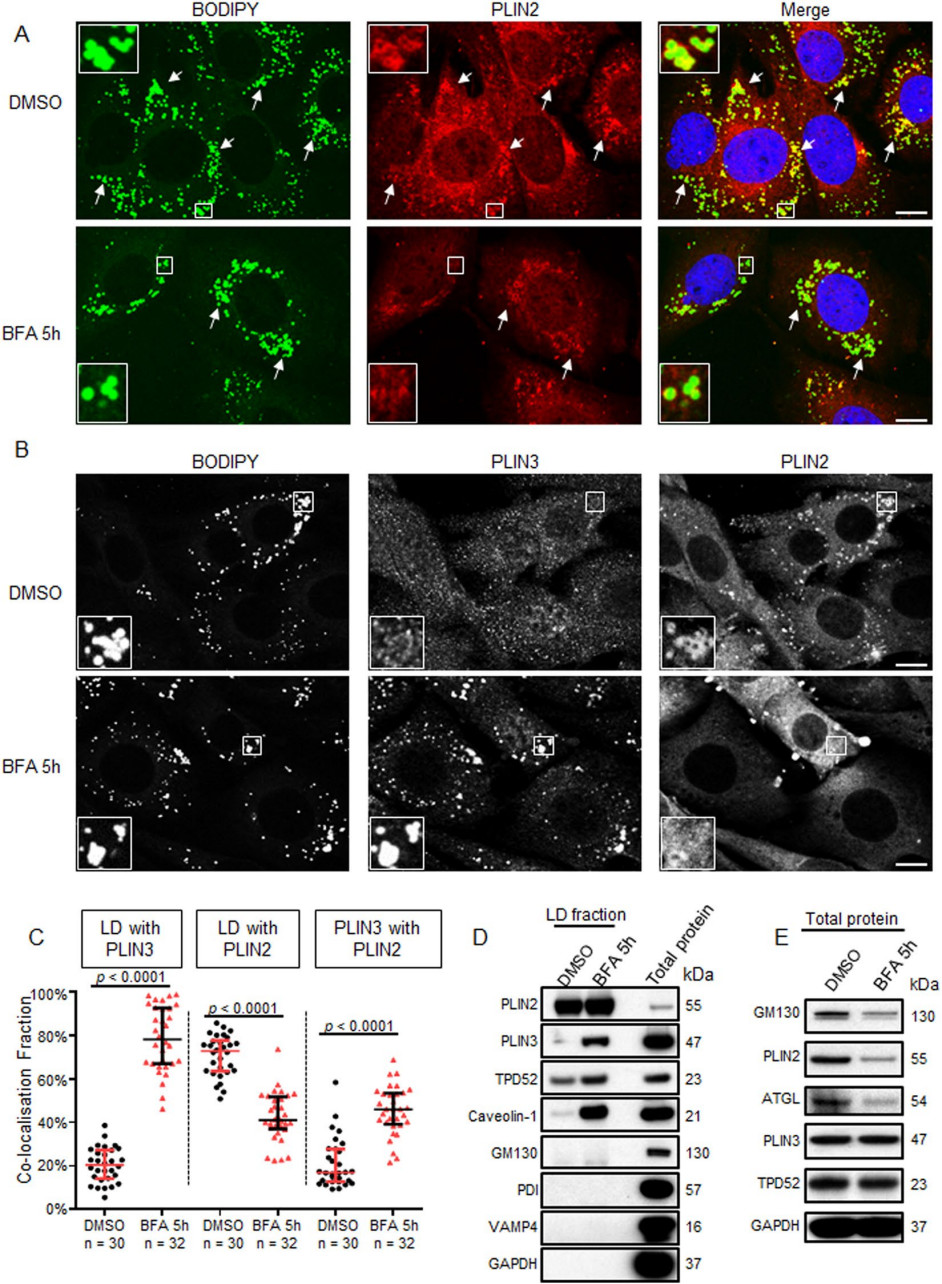
Recruitment of PLIN3 to LDs and loss of PLIN2 from LDs in BFA-treated D52-2-7 cells. (**A,B**) Immunofluorescence analyses of D52-2-7 cells treated with vehicle (DMSO) or 2 µg/ml BFA for 5 h, co-stained with (**A**) BODIPY (green), PLIN2 (red) and DAPI (blue), or (**B**) BODIPY, PLIN3 and PLIN2. Images are representative of those obtained in 3 independent experiments. Scale bar = 10 μm. (**A**) White arrows indicate PLIN2-associated LDs. Enlarged images of white boxed regions in vehicle-treated cells (DMSO) indicate LDs with PLIN2 staining, whereas enlarged images of the white boxed region in BFA-treated cells indicate LDs with limited PLIN2 staining. (**B**) Enlarged images of the white boxed region in vehicle-treated cells (DMSO) indicate LDs with PLIN2 staining but limited PLIN3 staining, whereas enlarged images of the white boxed region in BFA-treated cells indicate PLIN3-associated LDs with limited PLIN2 staining. (**C**) Quantification of Manders’ co-localisation coefficients (Y axis) between BODIPY-stained LDs and either PLIN3 (left) or PLIN2 (middle), and between PLIN3 and PLIN2 (right), quantified from the indicated numbers of images obtained from 3 independent experiments (below X axes) where cells were treated with either vehicle (DMSO, black circles) or BFA (red triangles) for 5 h. Horizontal lines indicate median values, bounded by interquartile ranges. *P* values, Mann Whitney *u* test. (**D**) LD fractions were purified from D52-2-7 cells treated with 400 μM OA complexed with fatty-acid-free BSA for 24 h, and then treated with DMSO or 2 µg/ml BFA for 5 h. Ten µg proteins extracted from LD fractions were analysed by Western blot and compared with 10 µg total proteins extracted from untreated D52-2-7 cells. (**E**) Western blot analyses of total protein extracts from D52-2-7 cells treated with DMSO or 2 µg/ml BFA for 5 h. Antisera to the proteins detected are shown at the left, and molecular weights (kDa) are shown at the right. GAPDH served as a loading control. See unprocessed Western blot images of (**D**,**E**) in Supplementary Fig. 11.

It has been reported that in AML12 (alpha mouse liver 12) cells incubated with oleic acid, PLIN3 was recruited to LDs following inhibition of COPI function, either by knockdown of COPI components or BFA treatment which phenocopied COPI knockdowns^19^. In contrast, BFA treatment or knockdown of COPI components in HeLa cells supplemented with oleic acid reduced ATGL and PLIN2 association with LDs, whereas the association of PLIN3 with LDs was unaffected^37^. We also noted that whereas almost all BODIPY-stained LDs were coated with PLIN2 in control D52-2-7 cells (Fig. 6A, DMSO), fewer LDs showed PLIN2 staining after 5 h BFA treatment (Fig. 6A, BFA 5 h). Furthermore, co-staining of PLIN3, PLIN2 and BODIPY revealed that, although PLIN3 showed limited detection on LDs in vehicle-treated D52-2-7 cells, 5 h BFA treatment markedly increased PLIN3 detection at LDs, while PLIN2 detection at LDs was significantly reduced (Fig. 6B). BFA treatment significantly increased LD and PLIN3 co-localisation by 3.8 fold, whereas the extent of co-localisation between LDs and PLIN2 nearly halved (Fig. 6C). BFA treatment also increased the co-localisation fraction between PLIN3 and PLIN2 by ~2.5 fold (Fig. 6C).

We further isolated LD fractions from D52-2-7 cells supplemented with 400 µM OA for 24 h and then treated with DMSO or 2 µg/ml BFA for 5 h. Western blot analyses indicated PLIN2 accumulation in LD fractions from DMSO-treated cells, with no reduction in PLIN2 levels after 5 h of BFA treatment, possibly because OA supplementation may have stabilised PLIN2 (Fig. 6D). In contrast, caveolin-1 levels were very low in LD fractions from DMSO-treated cells, but were significantly increased after 5 h BFA treatment (Fig. 6D), consistent with previous results^52^. Similarly, PLIN3 and TPD52 were detectable in LD fractions from DMSO-treated cells, and further increased upon BFA treatment (Fig. 6D). In contrast, GM130, PDI, VAMP4, and GAPDH were not detected in LD fractions from DMSO- or BFA-treated cells (Fig. 6D).

PLIN2 and ATGL are subjected to ubiquitin/proteasome degradation after dissociation from LDs^16,17,37^. Western blot analysis using total protein lysates extracted from DMSO- and BFA-treated D52-2-7 cells indicated that GM130, PLIN2, and ATGL levels decreased after 5 h BFA treatment, however no obvious changes were detected in PLIN3 or TPD52 levels (Fig. 6E).

### Differential dynamics of TPD52 and PLIN3 recruitment to LDs

**Figure 7:**
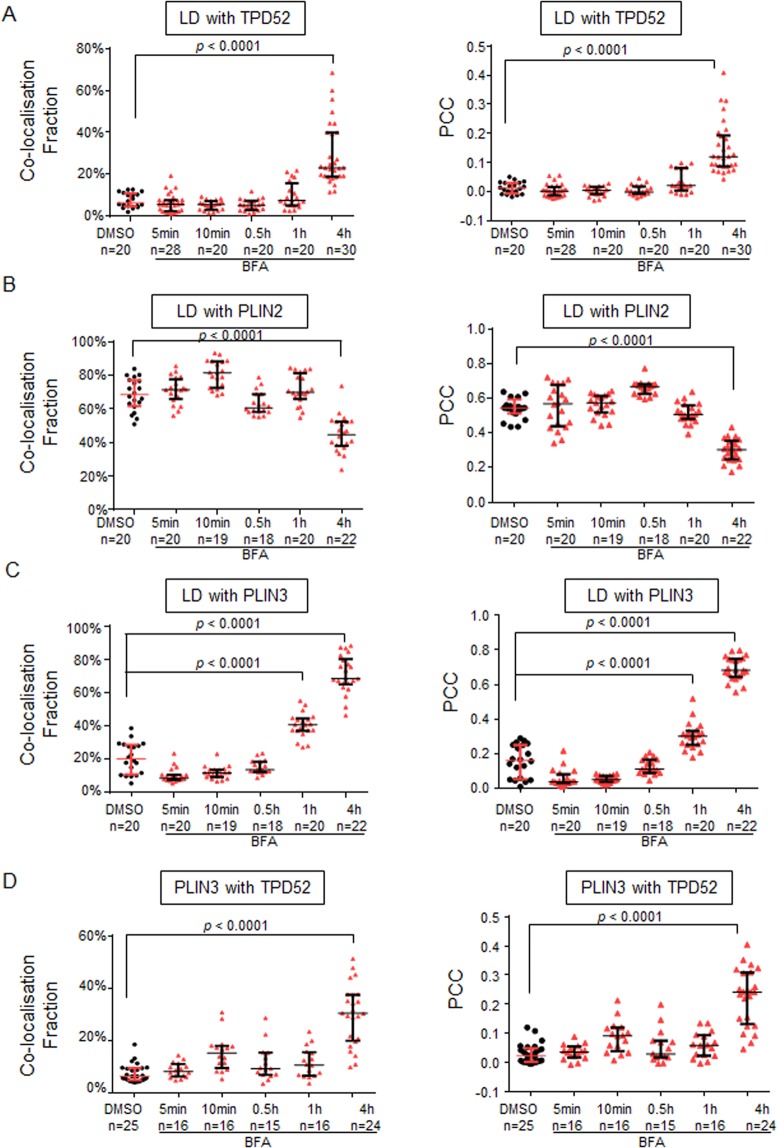
Differential dynamics of TPD52 and PLIN3 recruitment to LDs in D52-2-7 cells post-BFA treatment. Manders’ co-localisation coefficients (co-localisation fractions, Y axes, left) and Pearson’s correlation coefficients (Y axes, right) between (**A**) BODIPY-stained LDs and TPD52, (**B**) LDs and PLIN2, (**C**) LDs and PLIN3, and (**D**) PLIN3 and TPD52, quantified from the indicated numbers of images (below X axes) obtained from 3 independent experiments where cells were treated with either vehicle (DMSO, black circles) or BFA (red triangles) for the indicated time periods (X axes). Horizontal lines indicate median values, bounded by interquartile ranges. *P* values, Mann Whitney *u* test.

We sought to determine the dynamics of TPD52 and PLIN3 recruitment to LDs in D52-2-7 cells, compared with corresponding Golgi structural changes. TPD52 co-localised with GM130 at the juxta-nuclear region in control cells (Supplementary Fig. 5), with its perinuclear staining retained after 5 min-1 h BFA treatment. During this time, GM130 staining of an extended tubular network moved to dispersed punctate structures (Supplementary Fig. 5). However, after 4 h BFA treatment, a proportion of TPD52 clearly co-localised with LDs (Supplementary Fig. 5). PLIN3 was detected at LDs after 1 h BFA treatment, and co-localised with most LDs after 4 h BFA treatment, at which time PLIN2 detection at LDs was reduced (Supplementary Fig. 6). Quantification of extents of co-localisation showed significantly increased TPD52 detection at LDs after 4 h BFA treatment (Fig. 7A), whereas PLIN2 detection at LDs significantly decreased at this time point (Fig. 7B). In contrast, there was significant recruitment of PLIN3 to LDs after 1 h BFA treatment (Fig. 7C), whereas co-localisation between PLIN3 and TPD52 became prominent after 4 h BFA treatment (Fig. 7D).

### Recruitment of TPD52 to LDs is microtubule-dependent

The Golgi apparatus is a non-centrosomal microtubule-organising organelle^53,54^, and the effects of BFA on Golgi disassembly are microtubule-associated^27,42^. We therefore compared the effects of the microtubule-depolymerising agent nocodazole on the BFA-induced distributions of GM130, PLIN3 and TPD52.

**Figure 8:**
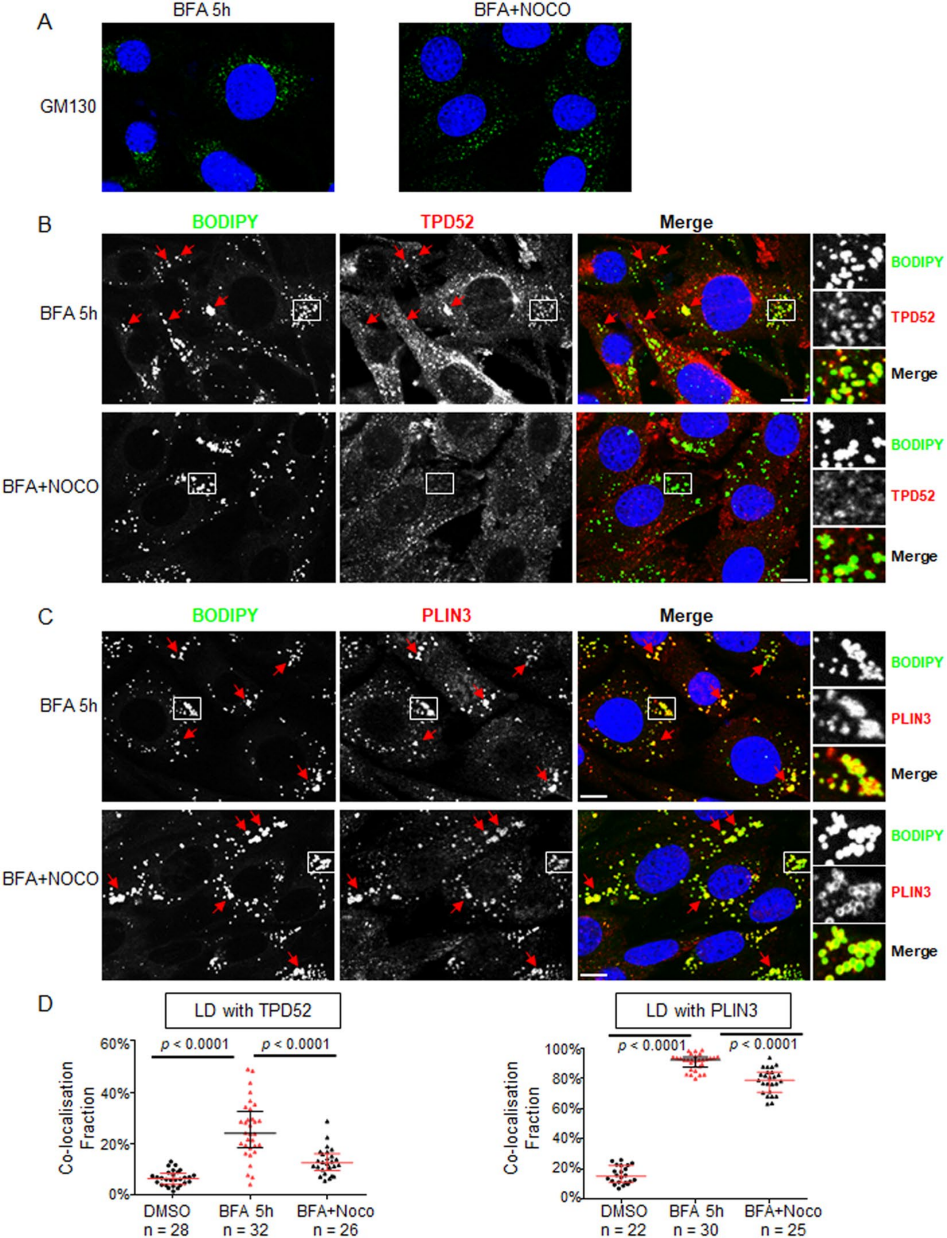
TPD52 recruitment to LDs in D52-2-7 cells is microtubule-dependent. D52-2-7 cells were treated with vehicle (DMSO) or 2 µg/ml BFA for 5 h (BFA 5 h), or with the addition of 2 µg/ml nocodazole after 4 h BFA treatment and incubation for another 1 h in the presence of BFA (BFA + NOCO), followed by immunofluorescent co-staining of (**A**) GM130 (green) and DAPI (blue), (**B**) TPD52 (red) and BODIPY (green), or (**C**) PLIN3 (red) and BODIPY (green). Enlarged images of white boxed regions in (**B**) indicate co-localisation of TPD52 and LD after 5 h BFA treatment, but more limited TPD52 and LD co-localisation after BFA and nocodazole co-treatment. In contrast, enlarged images of white boxed regions in (**C**) show PLIN3 and LD co-localisation following both treatments. Red arrows indicate other examples of (**B**) TPD52 and LD-colocalisations or (**C**) PLIN3 and LD-colocalisations. Images are representative of those obtained in 3 independent experiments. Scale bar = 10 μm. (**D**) Manders’ co-localisation coefficients (co-localisation fractions, Y axes) between BODIPY-stained LDs and TPD52 (left), or LDs and PLIN3 (right), quantified from the indicated numbers of images (below X axes) obtained from 3 independent experiments where cells were treated with either vehicle (DMSO, black circles), BFA (BFA 5 h, red triangles), or BFA followed by nocodazole (BFA + Noco, black triangles). Horizontal lines indicate median values, bounded by interquartile ranges. *P* values, Mann Whitney *u* test.

α-Tubulin staining indicated that D52-2-7 cells treated with 2 µg/ml (6.6 µM) nocodazole for 5 h showed highly disrupted cellular microtubule structures (data not shown). We treated D52-2-7 cells with 2 µg/ml BFA for 4 h before adding 2 µg/ml nocodazole for another 1 h in the continued presence of BFA. GM130-stained fragments were scattered uniformly through the cytoplasm in cells co-treated with BFA and nocodazole, whereas GM130 staining revealed dispersed punctate structures in cells treated with BFA only, similar to the previously described ERGIC distribution^40^ (Fig. 8A). Interestingly, TPD52 was also detected as fragments scattered in the cytoplasm after BFA and nocodazole co-treatment, and showed more limited co-localisation with LDs, compared with cells treated with BFA for 5 h only (Fig. 8B,D, left). In contrast, the majority of PLIN3 remained associated with LDs in the presence of nocodazole (Fig. 8C,D, right).

### HA-tagged TPD52 mutant aa 40–184 is exclusively targeted to LD

**Figure 9:**
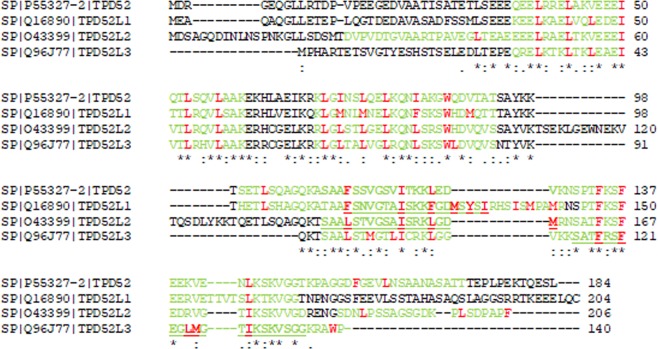
Predicted amphipathic helices and ALPS-like motifs in TPD52-like proteins. (**A**) Alignment of human TPD52, TPD52L1, TPD52L2 and TPD52L3 sequences (Swissprot isoform identifiers shown) using the one-letter code, produced by the Clustal Omega algorithm at EMBL-EBI. Numbers to the right of sequences refer to amino acid positions. Asterisks below the alignment indicate identical residues, and colons/dots indicate highly/weakly conserved residues scoring >0.5/≤0.5 in the Gonnet PAM 250 matrix, respectively. Hyphens within the alignment represent inserted gaps. Amphipathic helices predicted by HELIQUEST are shown in green, and ALPS-like motifs are shown in bold and underlined. Large hydrophobic residues (F, I, L, M, W, Y) within amphipathic helices are indicated in red.

**Figure 10:**
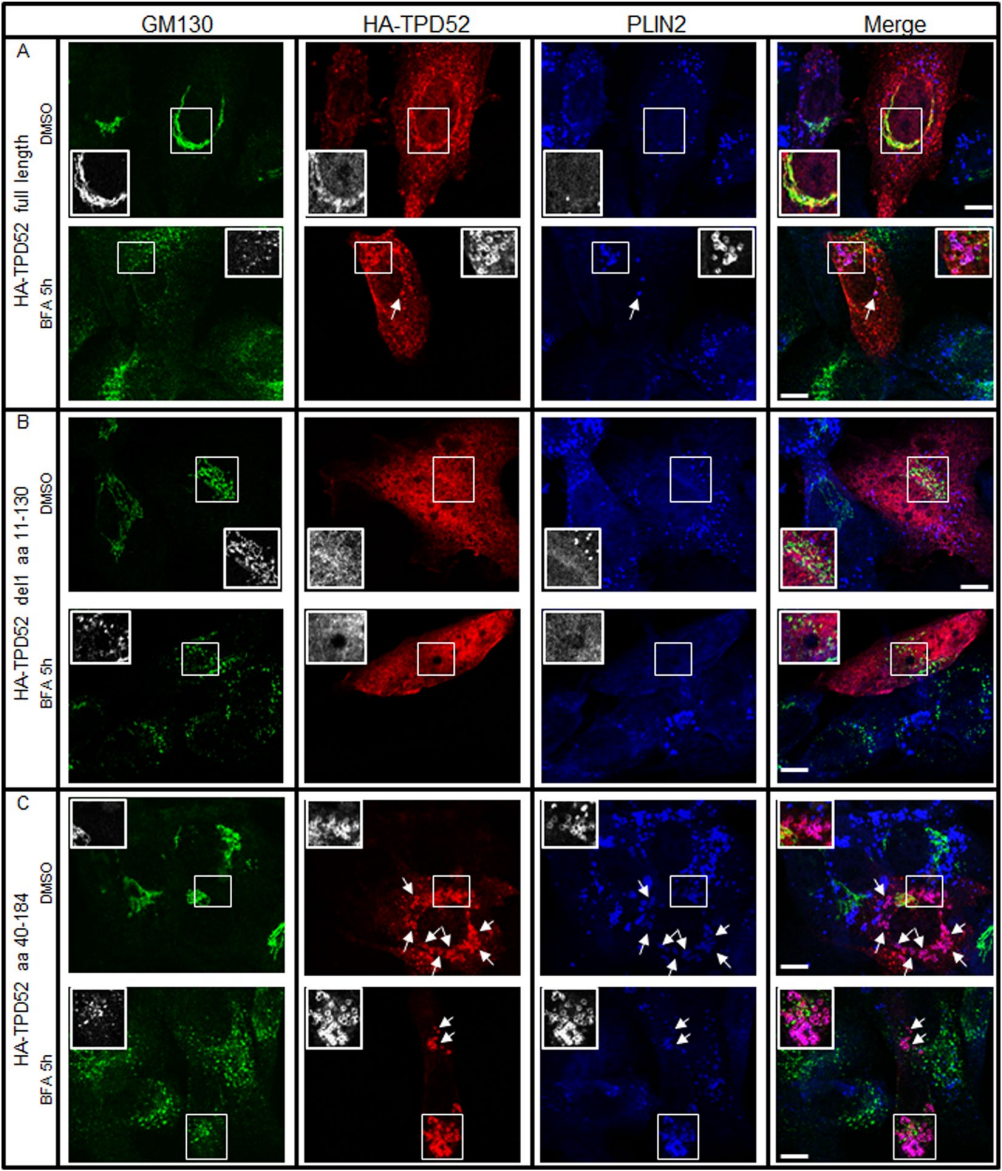
HA-tagged TPD52 aa 40–184 largely localised to LDs regardless of DMSO or BFA treatment. Immunofluorescence analyses of 3T3 cells transfected with (**A**) pHM6 *HA*-tagged full-length *TPD52*, (**B**) *HA-TPD52* del aa 111–130, (**C**) *HA-TPD52* aa 40–184 for 72 h and then treated with either vehicle (DMSO) or 2 µg/ml BFA (BFA) for 5 h, stained with GM130 (green), HA-TPD52 (red), and PLIN2 (blue). Enlarged images of white boxed regions show (**A**) co-localised HA-TPD52 and GM130 (DMSO), or co-localised HA-TPD52 and PLIN2 (BFA 5 h); (**B**) lack of colocalisation between HA-TPD52 del 111–130 aa and GM130, or PLIN2; (**C**) colocalisation between HA-TPD52 40–184 aa and PLIN2 in both DMSO and BFA treated cells. Other PLIN2- and TPD52-positive ring structures are indicated by white arrows. Images are representative of those obtained in 3 independent experiments. Scale bar = 10 μm.

Amphipathic helices are identified in PLIN proteins which bind directly to LD surfaces^8^. A general amphipathic α-helical motif, the ALPS (ArfGAP1 Lipid Packing sensor) motif first identified in ArfGAP1 mediates its binding to curved versus flat lipid membranes^55^. Using HELIQUEST^56^, amphipathic helices as well as ALPS-like motifs were predicted in members of the human TPD52 family (Fig. 9). TPD52 residues 111–128 fulfil most ALPS-like motif criteria, with the exception of the number of serine, threonine and glycine residues exceeding 6^56^ (Fig. 9). To further determine which TPD52 region(s) are responsible for Golgi- or LD-targeting, we generated a panel of HA-tagged TPD52 deletion constructs (Supplementary Fig. 7A) to test the subcellular localisations of these truncated proteins. Western blot analyses were performed to detect the levels of exogenously expressed TPD52 proteins in BALB/c 3T3 cells (Supplementary Fig. 7B). With a short Western blot exposure time (5 sec), HA-TPD52 del aa 111–130 and full-length HA-TPD52 were detected at comparable levels, whereas HA-TPD52 aa 1–111 and aa 1–131 levels were lower. HA-TPD52 aa 1–71 and aa 40–184 were detected after 30 min exposure, however HA-TPD52 aa 95–184 was poorly detected under these conditions and excluded from further analyses (Supplementary Fig. 7B). We then performed immunofluorescence analysis to compare the sub-cellular localisation of GM130, PLIN2, and HA-TPD52 mutants in transfected 3T3 cells treated with either 0.02% (v/v) DMSO or 2 µg/ml (7.1 µM) BFA for 5 h. Consistent with TPD52-stably expressing D52-2-7 cells, transient expression of HA-tagged full-length TPD52 in 3T3 cells showed co-distribution of HA-TPD52 with GM130 when treated with DMSO, and HA-TPD52 colocalised with PLIN2-stained LDs after 5 h BFA treatment (Fig. 10A). Unexpectedly, in contrast to all other truncated TPD52 proteins which showed ubiquitous cytoplasmic and nuclear staining (Fig. 10B and Supplementary Figs 8 and 9), the HA-tagged TPD52 aa 40–184 was almost exclusively detected on PLIN2-stained LDs, regardless of DMSO or BFA treatment (Fig. 10C and Supplementary Fig. 9).

## Discussion

Lipid droplets (LDs) are important cellular organelles involved in lipid metabolism as well as other processes such as response to ER stress, protein metabolism, and pathogen infection^2,3,5,57^. LDs are highly dynamic yet tightly regulated between lipid storage and mobilisation, which not only results in considerably varied numbers and sizes of LDs in different cell types and between individual cells, but also changes in the compositions of lipids and proteins at LDs^8,18,58^. We have previously identified that TPD52, a protein that is overexpressed in many different cancer types^32^, is detected at both the Golgi apparatus and LDs in TPD52-expressing 3T3 cells^34^. In this study, we examined the effects of BFA upon the sub-cellular localisation and LD recruitment of TPD52 and other LD regulators in TPD52-expressing 3T3 cells, and in human AU565 and HMC-1-8 breast cancer cells, all of which contain prominent LDs.

In our study, BFA treatment disrupted Golgi structures within minutes in D52-2-7 cells, as evidenced by the dispersed punctate staining of GM130, as previously reported^40^. However, TPD52 staining remained perinuclear after 5 min-1 h BFA treatment, and co-localised with syntaxin 6 at this region. After 1 h and 3 h BFA treatment, the TGN collapsed around the microtubule organising centre to form a dense juxtanuclear spot as previously reported^44,48^, and TPD52 clearly co-localised with syntaxin 6 at this site (Fig. 3C). As previous studies have shown that TPD52 regulates endo-lysosomal trafficking in secretory cell types^59–61^, our results suggest that the localisation of TPD52 at the Golgi/TGN may provide a link for cargo trafficking between TGN and endo-lysosomal systems.

BFA treatment inhibits the assembly of coatomer through inhibiting the activation of ARF family proteins by guanine nucleotide exchange factors (GEF)^21,22^. Knockdown of components of the ARF1/COPI complex dramatically increased lipid storage^19,20^, which may be partially due to decreased lipolysis caused by defective ARF1/COPI-dependent protein targeting to the LD^19,37^. BFA treatment, which phenocopies COPI knockdowns, also increases lipid storage in different systems including microalgae^62^, and mammalian cells^19,37^. In NRK and CHO cells, BFA stimulated mono-ADP-ribosylation and inactivation of CtBP1/BARs, a protein that is involved in transcriptional co-repression and Golgi membrane fission^63^. This resulted in the up-regulation of genes that regulate lipid storage and led to loss of LDs after 12 h BFA treatment, which was demonstrated to be independent of Golgi fragmentation and not associated with the unfolded protein response^64^.

In our study, although up to 5 h BFA treatment did not significantly alter triglyceride levels, BFA treatment significantly reduced LD numbers in D52-2-7 and HMC-1-8 cells, and increased LD sizes in D52-2-7, HMC-1-8 and AU565 cells. Furthermore, TPD52 knockdown in D52-2-7 cells not only decreased LD numbers and sizes in DMSO-treated cells, but also blunted BFA’s effects on LD numbers. Interestingly, five-hour BFA treatment induced a dramatic increase in TPD52 detection at LDs in D52-2-7 cells, with the median fraction of LDs associated with TPD52 increasing by approximately 4-fold, which was partially reversible upon BFA removal. Similar results were obtained in the breast cancer cell lines AU565 and HMC-1-8, with the median fractions of LDs associated with TPD52 increasing by ~2 fold in AU565 cells and by ~3 fold in HMC-1-8 cells after 5 h BFA treatment. These results indicate that TPD52 may play a role in lipid storage regulation induced by BFA treatment not only in TPD52-expressing 3T3 cells, but also when endogenously expressed in breast cancer cells that contain prominent LDs.

Similar timing of TPD52 recruitment to LDs was noted in 3T3 cells expressing TPD52 exogenously, and in human AU565 and HMC-1-8 cells expressing TPD52 endogenously. Whereas LD numbers in D52-2-7 cells were significantly reduced after 3 h BFA treatment, LD sizes gradually increased after 1–5 h BFA treatment. TPD52 detection at LDs only significantly increased after 4–5 h BFA treatment, which coincided with the significant loss of PLIN2 from LDs. The 2 breast cancer cell lines also showed significantly increased LD sizes 5 h post-BFA treatment, which was accompanied by significantly increased detection of TPD52 at LDs. The ARF1/COPI machinery may therefore limit the abundance of TPD52 at LDs under steady-state conditions. BFA treatment that inhibits ARF1/COPI machinery and induces LD expansion and/or clustering may increase the demand for other proteins to coat LDs, particularly when combined with PLIN2 loss. As significant TPD52 recruitment to LDs was only detected after 4–5 h BFA treatment, this indicates that TPD52 may not be responsible for the initial LD expansion, but rather responds to the presence of larger LDs.

The cell lines we examined differed in their expression of PLIN2 and PLIN3, and in the timing of PLIN3 recruitment to LDs following BFA treatment. TPD52-expressing 3T3 cells show relatively high PLIN2 levels^34^, whereas HMC-1-8 cells show low PLIN2 but higher PLIN3 levels. PLIN3 showed diffused cytoplasmic staining in D52-2-7 cells under routine culture conditions which may indicate PLIN3 association with nascent LDs. In D52-2-7 cells, PLIN3 was significantly recruited to LDs after 1 h BFA treatment, and co-localised with most LDs after 4 h BFA treatment. Western blot analyses of total proteins extracted from D52-2-7 cells after 5 h BFA treatment revealed reduced PLIN2 and ATGL levels, possibly due to protein degradation following their dissociation from LDs^16,17,37,65^. These results suggest that PLIN3 was recruited to maintain existing LDs. In contrast, in HMC-1-8 cells PLIN3 association with LDs was not significantly changed after 5 h BFA treatment, possibly because there was little PLIN2 to be lost from LDs in this cell line, and/or because there was already maximal association between PLIN3 and LDs in vehicle-treated cells. These results show incomplete concordance with those reported in other cell lines. In mouse AML12 cells incubated with oleic acid, PLIN3 was recruited to LDs following inhibition of COPI function (either by knockdown of COPI components or BFA treatment), whereas PLIN2 localisation remained unchanged^19^. In contrast, BFA treatment or knockdown of COPI components in HeLa cells supplemented with oleic acid decreased both ATGL and PLIN2 association with LDs, whereas PLIN3 association with LDs was unaffected^37^. These discrepancies may reflect differences in LD protein compositions in different cell types.

We have previously shown that TPD52 directly interacts with PLIN2 and PLIN3^34^. The fact that in D52-2-7 cells treated with BFA, PLIN3 is recruited to LDs prior to TPD52 recruitment could suggest that TPD52 is recruited to LDs through PLIN3 binding. However, BFA and nocodazole co-treatment resulted in TPD52 but not PLIN3 showing more limited co-localisation with LDs, compared with cells treated with BFA only. These findings suggest that the different timing of TPD52 versus PLIN3 recruitment to LDs in D52-2-7 cells reflected the fact that TPD52 but not PLIN3 trafficking was microtubule-dependent. Both TPD52 and caveolin-1 were detected at low levels in LD fractions from DMSO-treated D52-2-7 cells, but at higher levels in LD fractions after 5 h BFA treatment (Fig. 6D). Caveolin-1 recruitment to LDs has been proposed to reflect the microtubule-independent overflow of caveolins from the ER to LDs when ER-Golgi vesicle transport is blocked by BFA treatment^52,66,67^. However, since TPD52 neither co-localises with an ER marker^34^, nor possesses hydrophobic hairpin helices like caveolins^52,66,67^, TPD52 is likely to be recruited to LDs through alternative mechanism(s).

It is proposed that there are 2 major classes of proteins targeted to the LD surface. Class I proteins target the LD surface from the ER, typically through hydrophobic hairpin helices (e.g. GPAT4, caveolins), whereas class II proteins target LDs from the cytosol via domains such as amphipathic helices (e.g. PLIN proteins, CCT1)^6,8,57^. As a member of the LD-coating PLIN family, PLIN3 contains 11-mer helical repeats at the N-terminal region and a four-helix bundle at the C-terminal region that both contribute to LD targeting^8,68^. A general amphipathic α-helical motif, the ALPS (ArfGAP1 Lipid Packing sensor) motif, has been shown mediate ArfGAP1 binding with curved versus flat lipid membranes^55^. While unstructured in solution, the ALPS motif can place its large hydrophobic residues between loosely packed lipids and forms amphipathic helices with its abundant serine and threonine residues on the polar face^55,69^. TPD52L2, another member of the TPD52 family which has been reported as a LD-associated protein by proteomic studies^70,71^, has been predicted to contain an ALPS-like motif between residues 141–158 (Fig. 9)^72^. Using HELIQUEST^56^, amphipathic helices as well as ALPS-like motifs were predicted in members of the human TPD52 family (Fig. 9). Residues 111–128 within TPD52 fulfil most ALPS-like motif criteria, with the exception of the number of serine, threonine and glycine residues exceeding 6^56^ (Fig. 9). These predicted structural features suggest that TPD52 family proteins may have reversible lipid-binding capacity, which could explain the association of TPD52/TPD52L2 with LDs.

Recently, it has been proposed that amphipathic helices with large hydrophobic residues (I, F, L, M, W, Y) can detect and bind to large, persistent membrane packing defects, leading to LD targeting^15^. The LD binding index of amphipathic helices containing at least 6 large hydrophobic residues proportionally increases with the number of such residues^15^. TPD52 contains amphipathic helices with 5, 6 and 9 large hydrophobic residues in the first (aa 36–60), second (aa 70–93) and third (aa 100–172) amphipathic helix, respectively (Fig. 9). Interestingly, the deletion of C-terminal regions of TPD52 (constructs encoding aa 1–71, aa 1–111, or aa 1–131) abolished its localisation to either Golgi or LD. Furthermore, HA-tagged TPD52 lacking residues 111–130, which deleted the ALPS-like motif and 3 of the 9 large hydrophobic residues from the third helix, targeted neither LDs nor Golgi. In contrast, the N-terminally deleted mutant TPD52 aa 40–184 almost exclusively co-localised with PLIN2-stained LDs, regardless of DMSO or BFA treatment. These results suggest that the third TPD52 amphipathic helix which contains 9 large hydrophobic residues is necessary for LD targeting. Furthermore, the TPD52 residues 1–39 may serve as a negative regulator for LD-targeting. Our results are similar to those from studies performed using caveolin-1 deletion constructs where caveolin-1 with aa 46–95 deletion located to LDs regardless of BFA treatment^52,66^, although comparing the relevant TPD52 and caveolin-1 sequences revealed no obvious sequence similarity (data not shown). The N-terminus of TPD52 (residues 1–94) may also be important for preventing TPD52 from degradation, as the expression level of TPD52 aa 40–184 was very low compared to other C-terminally deleted mutants, and TPD52 aa 95–184 was barely detectable. As TPD52 contains PEST sequences at both N- and C-termini (Supplementary Fig. 7A) which could act as a signal peptide for protein degradation through the proteasome^73^, our results indicate that TPD52 aa 1–94 may be involved in maintaining the stability of the protein.

The comparatively delayed timing of TPD52 recruitment to LDs in response to BFA treatment, combined with our previous results showing increased TPD52 detection on LDs upon oleic acids supplementation^34^, suggests that TPD52 represents a late-responding protein that is recruited to LDs to stabilise LDs and/or to accommodate the increased demand for lipid droplet packaging induced by BFA treatment or lipid loading (Supplementary Fig. 10). This suggests the existence of a possible temporal hierarchy of proteins that are trafficked to LDs by distinct mechanisms in response to altered lipid storage requirements^57,58,74^. Comparatively late responding proteins such as TPD52 may function as a “second wave” of LD-recruited proteins, allowing finer tuning of lipid storage and/or the stable maintenance of LD phenotypes over time. Delayed recruitment of TPD52 to LDs may also reflect a preference for TPD52 to bind larger LDs, or to compete less effectively for LD binding than other LD-associated proteins. Although TPD52 has been reported to bind PLIN proteins^34^, the differential timing of PLIN2, PLIN3 and TPD52 recruitment to LDs that we have reported suggests that TPD52 does not rely upon binding to PLIN proteins for its recruitment to LDs. The delayed recruitment of LD-associated proteins by distinct mechanisms may provide redundancy if earlier response mechanisms are impaired or disrupted. Further studies of the timing of protein recruitment to LDs may identify other late-responding LD regulators, and clarify their roles in regulating lipid storage and metabolism.

## Materials and Methods

### Cell lines and cell culture

Human breast carcinoma cells HMC-1-8 (obtained from Creative Bioarray, Shirley, NY) and AU565^50^ were cultured in GIBCO^®^ RPMI 1640 (Thermo Fisher Scientific, VIC, AU) medium supplemented with 10% FBS (Thermo Fisher Scientific, VIC, AU) and 6 mM L-glutamine (Thermo Fisher Scientific, VIC, AU) at 37 °C with 5% CO_2_. The identity of AU565 was confirmed through short tandem repeat profiling by CellBank Australia (Westmead, NSW, AU). Vector-, and *TPD52*-transfected stable BALB/c 3T3 cell lines have been previously reported^39^, and were cultured in GIBCO^®^ RPMI 1640 medium supplemented with 10% FBS and 6 mM L-glutamine, with the addition of 1 mg/mL G418 (Geneticin®, Thermo Fisher Scientific, VIC, AU).

### Plasmids

The pHM6 *HA*-tagged *TPD52* (full-length aa 1-184, NM_005079.3, transcript variant 3) and control vectors have been previously described^34,60^. The pHM6 *HA*-tagged *TPD52* aa 1–71, aa 1–111, aa 1–131, aa 40–184, and aa 95–184 constructs were generated by ligating PCR amplified *TPD52* cDNAs digested with *Hind*III and *Kpn*I into the same sites of the pHM6 plasmid. PCR amplification was carried out using *Pfx*50 DNA polymerase (Thermo Fisher Scientific, VIC, AU) and primers described in Supplementary Table 1. The deletion of TPD52 aa 111–130 was firstly generated by digesting pGEX-6P-1 full-length *TPD52*^34^ with *Stu*I and *Xho*I, giving rise to the fragment encoding aa 1–110, and then ligating with the PCR amplified aa 131–184 fragment (primers shown in Supplementary Table 1) digested with *Stu*I and *Xho*I. The pGEX-6P-1 *TPD52* del aa 111–130 then served as a template to amplify the *TPD52* cDNA with aa 111–130 deletion using primers described in Supplementary Table 1, which was digested with *Hind*III and *Kpn*I, and then subcloned into the same sites of pHM6. All constructs were validated by Sanger sequencing [Australian Genome Research Facility (AGRF), NSW, AU].

### Reagents

Brefeldin A (BFA, Sigma Aldrich, NSW, AU) and Nocodazole (Sigma Aldrich, NSW, AU) were reconstituted in DMSO as 10 mg/ml stock solutions. BODIPY 493/503 (Thermo Fisher Scientific, VIC, AU) was reconstituted in ethanol as a 1 mg/ml stock solution.

### Antibodies

Affinity-purified rabbit polyclonal TPD52 antisera (1:100 for immunofluorescence, IF)^75^ and TPD52 mouse monoclonal antibody (1:10 for IF)^76^ have been described previously. TPD52 rabbit monoclonal antibody (EPR14219, 1:250 for IF and 1:2000 for Western blot, WB) was purchased from Abcam (VIC, AU). GM130 mouse monoclonal antibody (35/GM130, 1:1,000 for both IF and WB) was purchased from BD Transduction Laboratories (San Jose, CA). Adipophilin/PLIN2 guinea pig polyclonal antibody (1:200 for IF and 1:2000 for WB, Progen Biotechnik, Heidelberg, Germany) was used to detect mouse PLIN2, whereas sheep anti-human PLIN2 antibody (1:200 for IF and 1:1000 for WB) was a gift from Dr Enoch Tay (Westmead Institute for Medical Research, NSW, AU)^77^. PLIN3 guinea pig polyclonal antibody (1:200 for IF and 1:1000 for WB, Progen Biotechnik, Heidelberg, Germany) was used to detect human PLIN3, while PLIN3 rabbit polyclonal antibody (1:200 for IF and 1:1000 for WB, Proteintech, Rosemont, IL) was used to detect mouse PLIN3. Mouse monoclonal anti-α-tubulin (DM1A, 1:500 for IF) was purchased from Sigma-Aldrich (VIC, AU). Anti-adipose triglyceride lipase (ATGL) rabbit monoclonal antibody [EPR3444(2), 1:1000 for WB] was purchased from Abcam (VIC, AU). Syntaxin 6 rabbit monoclonal antibody (C34B2, 1:100 for IF), rabbit monoclonal anti-HA antibody (C29F4, 1:1600 for IF and 1:1000 for WB), ERBB2 rabbit monoclonal antibody (29D8, 1:1000 for WB), caveolin-1 rabbit polyclonal antibody (1:1000 for WB), and PDI rabbit monoclonal antibody (C81H6, 1:1000 for WB) were purchased from Cell Signaling Technology (Danvers, MA). VAMP4 polyclonal rabbit antibody (1:1000 for WB) was from Synaptic Systems GmbH (Gottingen, Germany). Mouse monoclonal glyceraldehyde 3-phosphate dehydrogenase (GAPDH) antibody (6C5, 1:10,000, Thermo Fisher Scientific, VIC, AU) or mouse monoclonal β-actin antibody (AC-74, 1:5,000, Sigma Aldrich, NSW, AU) were used as loading controls in Western blot analyses.

Alexa Fluor®488 anti-mouse IgG (2 µg/mL, Thermo Fisher Scientific, VIC, AU), Cy3-conjugated anti-rabbit IgG (1.5 µg/mL) and Cy3-conjugated anti-sheep IgG (1.2 µg/mL, Jackson ImmunoResearch Laboratories Inc, West Grove, PA), Alexa Fluor®633 anti-guinea pig IgG (2 µg/mL) and Alexa Fluor^®^633 anti-mouse IgG (2 µg/mL, Thermo Fisher Scientific, VIC, AU) were utilised as secondary antibodies in immunofluorescence analyses at 1:1000 dilution.

### Triglyceride assays

Cellular total lipid was extracted using the Folch method^78^ and triglyceride concentrations were measured using a GPO-PAP kit (Roche Diagnostics, NSW, AU) according to the manufacturer’s instructions, and normalised according to cellular protein levels^34^.

### Isolation of lipid droplets by cellular fractionation

Lipid droplet fractionation was carried out as previously described^34,79^. Briefly, D52-2-7 cells were treated with 400 μM oleic acid complexed to fatty acid-free BSA for 24 h, followed by treatment with 2 µg/ml (7.1 µM) BFA, or vehicle only (0.02% (v/v) DMSO) for 5 h, and then lysed in hypotonic lysis buffer^34,79^. The lysates were briefly sonicated and applied to a sucrose gradient, centrifuged at 28,000 g in a Beckman SW41Ti rotor for 45 min at 4 °C, and the lipid droplet fraction was collected from the top layer. Proteins from the lipid droplet fraction were precipitated with 20% trichloroacetic acid (TCA, Sigma-Aldrich, NSW, AU) followed by acetone wash. After air drying, the pellet was dissolved in lysis buffer [150 mM NaCl, 5 mM EDTA, 50 mM Tris⋅HCl pH 7.5, 1% Triton X-100, 50 mM sodium fluoride, 1 mM sodium orthovanadate, 1 mM phenylmethylsulfonyl fluoride (PMSF), and EDTA-free Protease Inhibitor Cocktail Tablets]. Protein concentrations were measured using a Pierce™ BCA Protein Assay Kit (Thermo Scientific, VIC, AU), and 10 μg protein was subjected to Western blot analyses^34,79^.

### Transient small interfering RNA transfection

The *TPD52* siRNA duplex (5′-GCGGAAACUUGGAAUCAAU-3′) as described previously^38,39^, was synthesised by Dharmacon RNAi Technologies (Thermo Fisher Scientific, VIC, AU). Non-targeting siRNA (Allstar Negative Control siRNA) was purchased from QIAGEN (VIC, AU).

TPD52-expressing BALB/c 3T3 cell line D52-2-7 cells were plated into 24-well plates, or onto glass coverslips in 6-well plates. After 24 h incubation, cells were transfected with 50 nM siRNAs using *Trans*IT-TKO transfection reagent (Mirus, Madison, WI) in complete media according to the manufacturer’s instructions^38^. At 72 h post transfection, cells were treated with 2 µg/ml (7.1 µM) BFA, or with vehicle only (0.02% (v/v) DMSO) for 5 h, and subjected to further analyses.

### Transient plasmid transfection

BALB/c 3T3 cells were seeded into 12-well plates 24 h prior to transfection. Transfections were performed by adding pHM6 *HA-LacZ*, pHM6 full-length *HA-TPD52*, or pHM6 *HA-TPD52* deletion constructs with *Trans*IT-X2 transfection reagent (Mirus, Madison, WI) at a ratio of 1:3, according to the manufacturer’s instructions^38^. Cells were maintained at 37 °C in a 5% CO_2_ humidified atmosphere for 72 h before further analyses.

### Indirect immunofluorescence analyses

Cells were treated with 2 µg/ml (7.1 µM) BFA, or vehicle only (0.02% (v/v) DMSO) for the indicated time periods at 37 °C, or cells were treated with 2 µg/ml (7.1 µM) BFA for 4 h, then 2 µg/ml (6.6 µM) nocodozale was added and incubated for another 1 h in the continued presence of BFA. In some experiments, cells were washed with PBS after 5 h BFA treatment and allowed to recover for 1 h in complete growth media. For LD quantification, cells were fixed in 3% (w/v) formaldehyde in PBS for 30 min at RT, then stained with 1 µg/ml BODIPY 493/503 (Life technologies, VIC, AU) for 30 min at RT. DNA was counterstained using 4′,6-diamidino-2-phenylindole, dihydrochloride (DAPI, Sigma-Aldrich, NSW, AU; 1 µg/mL)^34^. For co-staining of LD and proteins, cells were fixed with 3% (w/v) formaldehyde and 0.025% (v/v) glutaraldehyde in PBS for 10 min at RT, quenched with 50 mM ammonium chloride in PBS for 10 min, and permeabilised using 0.1% Triton X-100 in PBS^34,80^. Cells were then stained with primary antibodies diluted in 3% (w/v) BSA/PBS overnight at 4 °C, followed by incubation in secondary antibodies diluted in 3% BSA/PBS for 1 h at RT. BODIPY 493/503 solution was then added to a final concentration of 10 µg/ml in PBS and applied for 30 min, followed by DNA counterstaining using DAPI. For other indirect immunofluorescence analyses, cells with indicated treatments were fixed in 3% (w/v) formaldehyde in PBS for 15 min at RT, then in cold acetone: methanol (v/v, 1:1) for 15 min at −20 °C, followed by primary and secondary antibodies incubation as described above^34^. All samples were viewed with a Leica TCS SP5 II confocal microscope (NSW, AU) using a 63× objective lens unless otherwise stated.

### Image analyses

Cellular lipid droplet quantification was performed as previously described using Image-Pro Plus Version 7.0 software (MediaCybernetics, Rockville, MD)^34^. Objects with diameters smaller than 0.3 μm were filtered to reduce noise from potential non-specific staining. The intensity range was set according to the BODIPY 493/503 fluorescence intensity detected in one TPD52-expressing cell line, D52-2-7, and the same parameters were then used to quantify LDs in all cell lines^34^.

Degrees of co-localisation were measured between LDs and TPD52/PLIN2/PLIN3, or between TPD52 and syntaxin 6/PLIN2/PLIN3. Image analysis was undertaken with a bespoke script written in MATLAB (MathWorks, USA) (Supplementary information). Images were separated into red, green, and blue channels and pre-processed by median and Wiener filters^81^. Signal location was identified using the MatCol algorithm^82^. Briefly, the algorithm identified signal locations where the luminance exceeds a threshold value, T, calculated as: T = μ + 2σ where μ is the mean and σ is the standard deviation of pixel intensities in the image. RGB signal co-localisations were calculated by Manders’ co-localisation coefficients (M1 and M2, which represent the fraction of total probe fluorescence that co-localises with the fluorescence of a second probe^83^) and Pearson’s correlation coefficients (PCC)^83^.

### Stimulated emission depletion (STED) microscopy

D52-2-7 cells were treated with 2 µg/ml (7.1 µM) BFA, or vehicle only (0.02% (v/v) DMSO) for 5 h at 37 °C, fixed in 3% (w/v) formaldehyde in PBS for 15 min at RT, then in cold acetone/methanol (v/v, 1:1) for 15 min at −20 °C. Cells were then stained by TPD52 rabbit monoclonal Ab (1:100) and Adipophilin/PLIN2 guinea pig polyclonal antibody (1:100) diluted in 3% (w/v) BSA/PBS overnight at 4 °C. Alexa Fluor®594 anti-rabbit IgG (2 µg/mL, Thermo Fisher Scientific, VIC, AU) and Alexa Fluor^®^633 anti- guinea pig IgG (2 µg/mL, Thermo Fisher Scientific, VIC, AU) were utilised as secondary antibodies at 1:200 dilution, and coverslips were mounted onto slides using Prolong Diamond Antifade Mountant (Thermo Fisher Scientific, VIC, AU). STED microscopy^84^ was performed on a Leica TCS SP8 STED 3X microsystem, located in Australian Microscopy & Microanalysis Research Facility at the Australian Centre for Microscopy & Microanalysis at the University of Sydney. The system was equipped with a pulsed white light excitation laser (470–670 nm), and 592 nm and 775 nm lasers for fluorophore depletion by STED. Images were taken using a HC PL APO 100×/1.4 oil STED WHITE objective lens. Imaging and setup of scans, including STED alignment, were performed using LAS AF software (Leica, NSW, AU).

### Western blot analyses

Cells were lysed in 3% SDS lysis buffer or NETN lysis buffer (150 mM NaCl, 5 mM EDTA, 50 mM Tris-HCl pH 7.5, 0.5% (v/v) Nonidet P-40) containing phosphatase and protease inhibitors (50 mM sodium fluoride, 1 mM sodium orthovanadate, 1 mM PMSF, and EDTA-free Protease Inhibitor Cocktail Tablets from Roche Applied Science)^34,38^. Ten μg total protein extracts were resolved by SDS-PAGE on NuPAGE^®^ Novex 4–12% Bis-Tris mini gels (Thermo Fisher Scientific, VIC, AU). Protein levels were assessed using FIJI ImageJ 1.52n freeware to quantify fold changes in TPD52 protein levels relative to GAPDH, which were then normalized to the ratio in cells treated with DMSO and non-targeting siRNA.

### Sequence analyses

Alignments between multiple protein sequences were produced by the Clustal Omega algorithm (EMBL-EBI). The degree of amino acid conservation was scored according to the Gonnet PAM 250 matrix. Amphipathic helices and ALPS motifs were predicted using the HELIQUEST algorithm http://heliquest.ipmc.cnrs.fr/56, followed by visual sequence inspection in the case of ALPS motifs^72^.

### Statistical analyses

Both SPSS for Windows (version 22; IBM) and GraphPad Prism 7 (GraphPad Software, La Jolla, CA) were used for graph generation and statistical analyses. The Mann Whitney *u* test was utilised to compare the median numbers of LDs per cell, areas per LD object, and total LD areas per cell, quantified from each image. The comparison of percentages of LD > 1 µm^2^ or ≤1 µm^2^ was made using Pearson’s Chi-Squared test. Pearson’s correlation coefficients (PCC) and Manders’ co-localisation coefficients between groups were also compared using the Mann Whitney *u* test. Values from triglyceride analyses were expressed as means ± s.e.m. of 3 independent experiments, and compared between groups using a two-tailed, unequal variance Student’s *t* test.

## Supplementary information

Supplementary information

Matlab source code
