# Supplementary material for: Delayed recruiting of TPD52 to lipid droplets – evidence for a “second wave” of lipid droplet-associated proteins that respond to altered lipid storage induced by Brefeldin A treatment
Source: Sci Rep. 2019 Jul 5;9:9790. doi: 10.1038/s41598-019-46156-1 (PMC6611826; doi:10.1038/s41598-019-46156-1)
Supplement: Supplementary file 1 — Supplementary information [file 41598_2019_46156_MOESM1_ESM.pdf]

# Supplementary Information

**Delayed recruiting of TPD52 to lipid droplets – evidence for a "second wave" of lipid droplet-associated proteins that respond to altered lipid storage induced by Brefeldin A treatment**

Yuyan Chen[1,2,#], Sarah Frost[1,2], Matloob Khushi[3], Laurence C. Cantrill[2,4], Hong Yu[5], Jonathan W. Arthur[3], Robert K. Bright[6], Guy E. Groblewski[7], Jennifer A. Byrne[1,2,#]

[1]Molecular Oncology Laboratory, Children's Cancer Research Unit, Kids Research The Children's Hospital at Westmead, Westmead, NSW 2145, Australia

[2]Discipline of Child and Adolescent Health, The University of Sydney, The Children's Hospital at Westmead, Westmead, NSW 2145, Australia

[3]Bioinformatics Unit, Children's Medical Research Institute, The University of Sydney, Westmead, NSW 2145, Australia

[4]Kids Research Microscope Facility, The Children's Hospital at Westmead, Westmead, NSW 2145, Australia

[5]Cell Imaging Facility, Westmead Institute for Medical Research, Westmead, NSW 2145, Australia

[6]Department of Immunology and Molecular Microbiology and TTUHSC Cancer Center, Texas Tech University Health Sciences Center, Lubbock, Texas 79430, USA

[7]Department of Nutritional Sciences, University of Wisconsin, Madison, Wisconsin 53706, USA

Supplementary Table 1. Primers used for PCR amplification to generate *TPD52* (NM_005079.3) deletion constructs.

| Encoded Fragment | Forward Primer (5'→3') | Reverse Primer (5'→3') |
|---|---|---|
| aa 1-71 | AAATAAGCTTCATGGACCGCGGCGA | GCATGGTACCTCAAAGTTTCCGCT |
| aa 1-111 | AAATAAGCTTCATGGACCGCGGCGA | GTATGGTACCTCATGAGGCCTTCT |
| aa 1-131 | AAATAAGCTTCATGGACCGCGGCGA | CGCCGGTACCTCAGGAGTTTTTTA |
| aa 40-184 | CGCCAAGCTTCATGAGAAGAGAACTT | ATTTGGTACCTCACAGGCTCTCCTG |
| aa 95-184 | CGCCAAGCTTCATGGCTTACAAGAAG | ATTTGGTACCTCACAGGCTCTCCTG |
| aa 131-184 | CTACAGGCCTCCCCAACTTTTA | ATTTCTCGAGTCACAGGCTCTC |
| del aa 111-130 | AAATAAGCTTCATGGACCGCGGCGA | ATTTGGTACCTCACAGGCTCTCCTG |

## Supplementary Figure 1

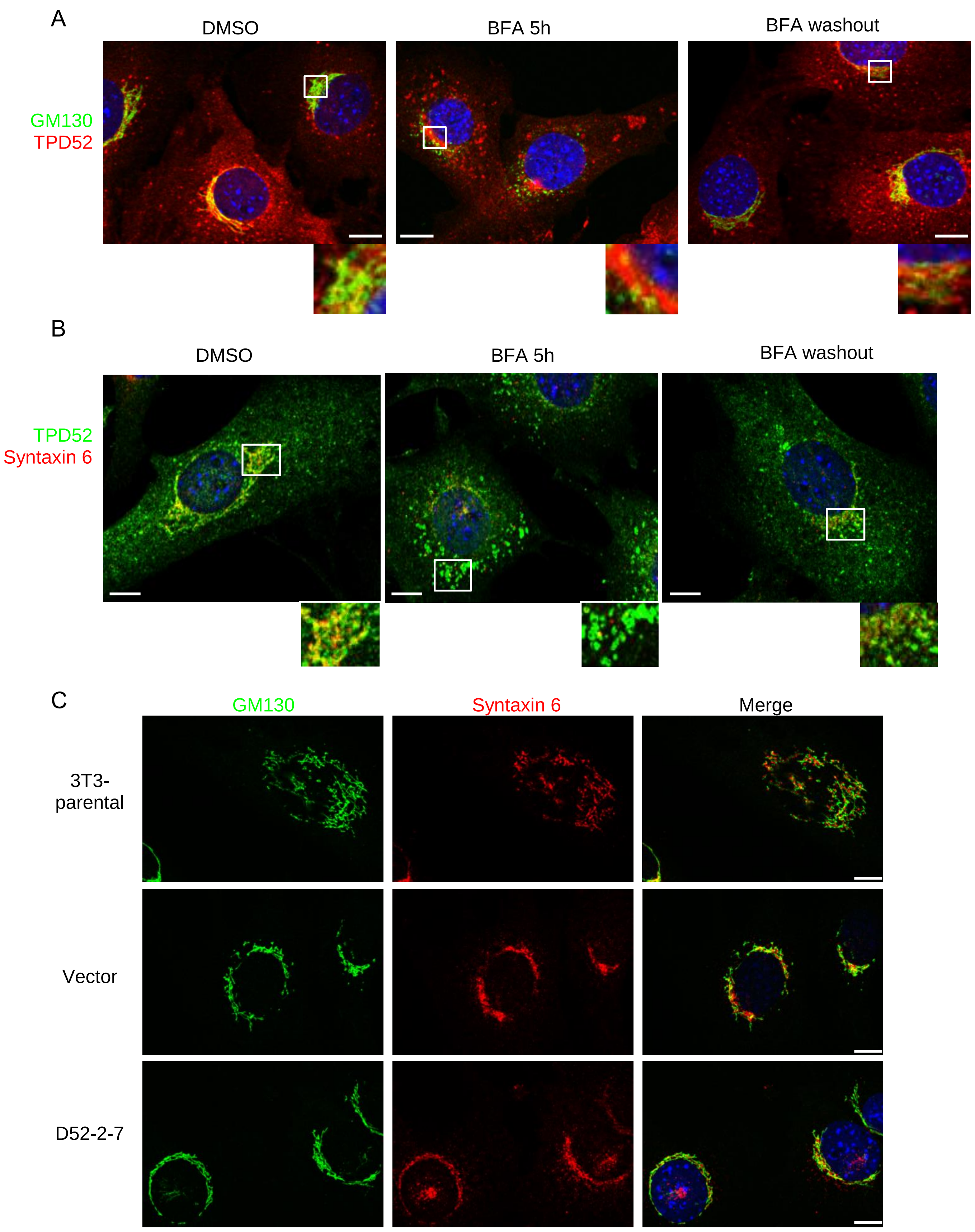

Suppl. Fig. 1. The BFA-induced redistribution of TPD52, GM130, and syntaxin 6 were reversible upon BFA washout. Immunofluorescence co-staining of (A) GM130 (green) and TPD52 (red), (B) syntaxin 6 (red) and TPD52 (green) in D52-2-7 cells treated with vehicle (DMSO) or 2 µg/ml BFA for 5 h, or following PBS washout after 5 h BFA treatment, and incubation at 37°C for 1 h in complete media without BFA (BFA washout). Enlarged images of white boxed regions show the distributions of TPD52 and GM130, or TPD52 and syntaxin 6 at perinuclear regions. Images are representative of those obtained in 3 independent experiments. (C) Immunofluorescence analyses of parental, vector-, and TPD52-transfected (D52-2-7) 3T3 cells, stained with GM130 (green), syntaxin 6 (red), and DAPI (blue), with merged images shown at right. Images are representative of those obtained in 3 independent experiments. Scale bar = 10 µm.

**Supplementary Figure 2**

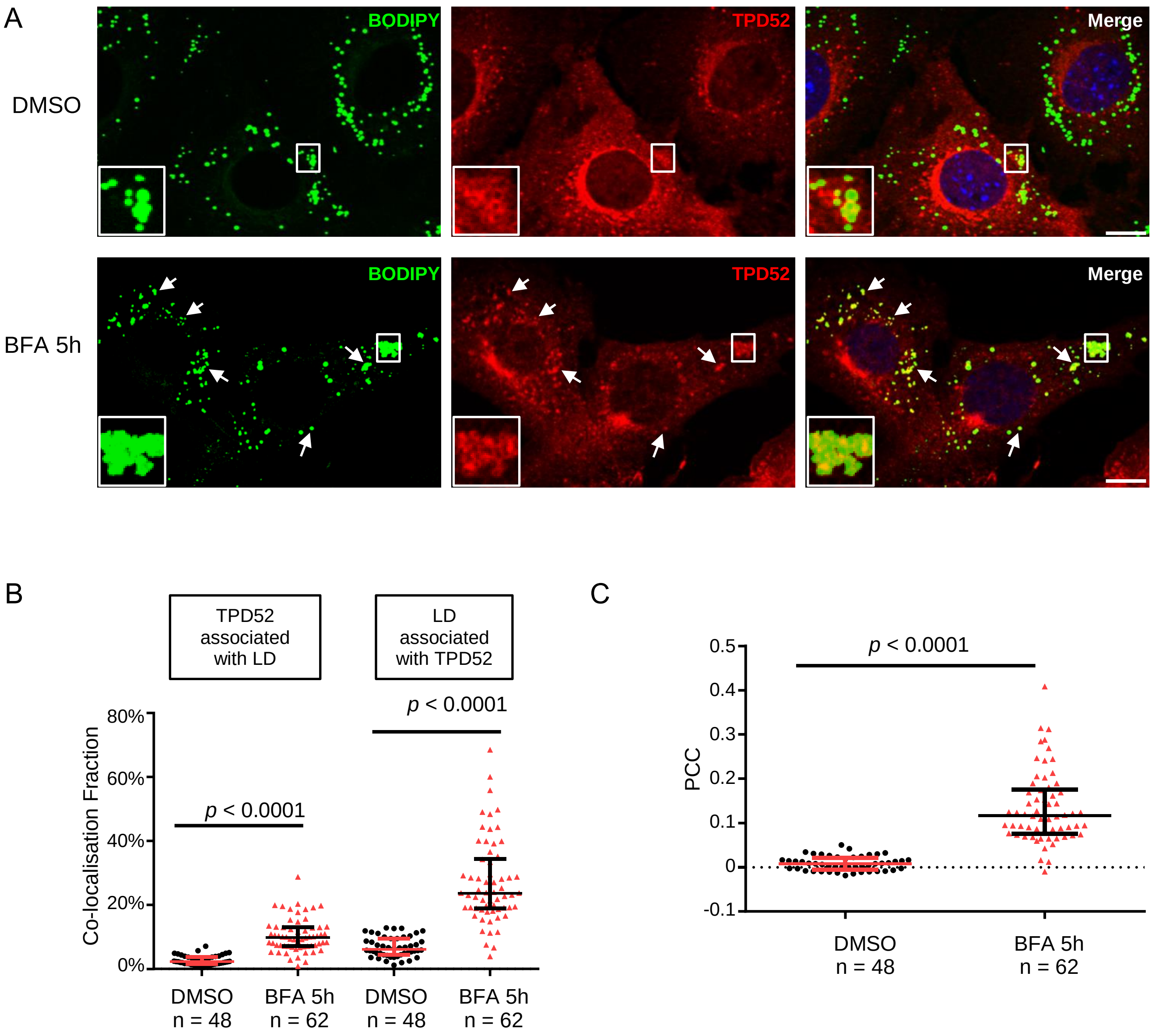


Suppl. Fig. 2. Significantly increased TPD52 detection at LDs post-BFA treatment in D52-2-7 cells. (A) Immunofluorescence analyses of D52-2-7 cells treated with vehicle (DMSO) or 2 µg/ml BFA for 5 h, stained with TPD52 (red), BODIPY (green) for LDs, and DAPI (blue) for nuclei. Enlarged images of white boxed regions indicate limited co-localisation between BODIPY and TPD52 staining in vehicle-treated cells (DMSO), which became more prominent after BFA treatment. White arrows indicate TPD52-positive LDs in BFA-treated cells. Images are representative of those obtained in 3 independent experiments. Scale bar = 10 µm. Quantification of (B) co-localisation fractions using Manders' co-localisation coefficients (Y axis) and (C) Pearson's correlation coefficients (PCC, Y axis) between BODIPY and TPD52 from the indicated numbers of images (below X axes) obtained from 3 independent experiments where cells were treated with vehicle (DMSO, black circles) or BFA for 5 h (red triangles). Horizontal lines indicate median values, bounded by interquartile ranges. *P* values, Mann Whitney *u* test.

## Supplementary Figure 3

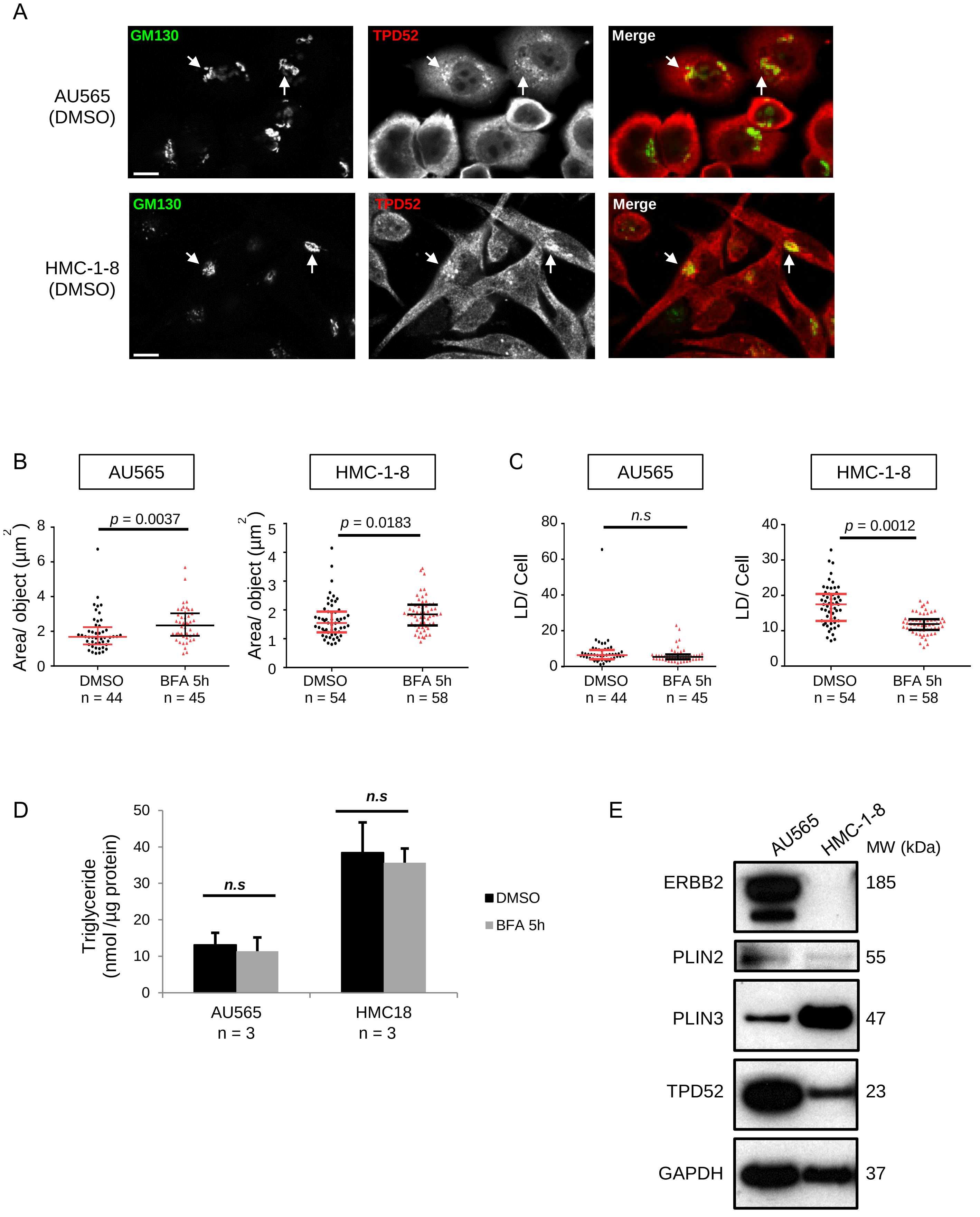

Suppl. Fig. 3. Effects of BFA treatment detected in AU565 and HMC-1-8 cells. (A) Immunofluorescence analyses of AU565 and HMC-1-8 cells stained with TPD52 (red), GM130 (green), with merged images. White arrows indicate the co-distribution of TPD52 and GM130. Scale bar = 10 μm. Quantification of (B) LD areas ($\mu m^2$) /object (Y axes), and (C) LD numbers /cell (Y axes) from the indicated numbers of images (below X axes) obtained from 3 independent experiments where AU565 or HMC-1-8 cells were treated with vehicle (DMSO, black circles) or BFA for 5 h (red triangles). Horizontal lines indicate median values, bounded by interquartile range. *P* values, Mann Whitney *u* test. n.s = not statistically significant. (D) Triglyceride levels (Y axis, nmol/ μg protein, mean values +/- s.e.m values from 3 independent experiments) measured in 5 h vehicle (black) or BFA-treated (grey) cells. n.s = not statistically significant, Student's t-test. (E) Western blot analyses of total AU565 and HMC-1-8 protein extracts using antisera to ERBB2, TPD52, PLIN2, and PLIN3, with molecular weights (MW) shown in kDa. GAPDH served as a loading control. See unprocessed Western blots in Supplementary Fig 11.

## Supplementary Figure 4

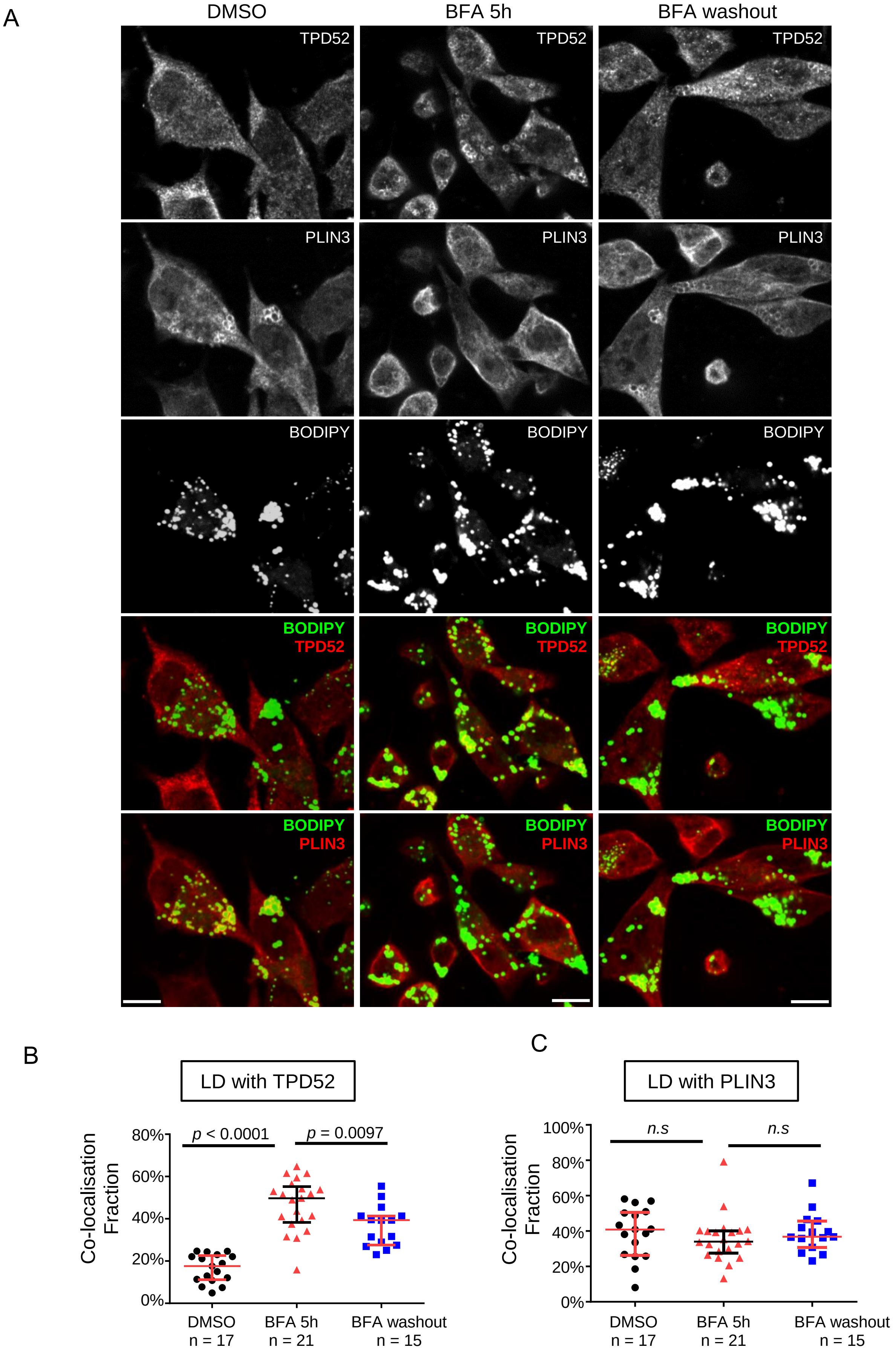

Suppl. Fig. 4. TPD52 detection increased at LDs post-BFA treatment in HMC-1-8 cells, which was partially reversed after BFA washout. (A) Immunofluorescence analyses of HMC-1-8 cells treated with vehicle (DMSO) or 2 µg/ml BFA for 5 h, or washed with PBS after 5 h BFA treatment and allowed 1 h recovery in complete media without BFA (BFA washout). Cells were co-stained with BODIPY, TPD52 and PLIN3, with merged images shown of BODIPY (green) and TPD52 (red), or BODIPY (green) and PLIN3 (pseudo-coloured red). Images are representative of those obtained in 3 independent experiments. Scale bar = 10 µm. Co-localisation fractions (Manders' co-localisation coefficients, Y axes) between (B) BODIPY-stained LDs and TPD52, or (C) BODIPY-stained LDs and PLIN3 (right) quantified from the indicated numbers of images (below X axes) obtained from 3 independent experiments where HMC-1-8 cells were treated with either vehicle (DMSO, black circles), BFA (BFA 5h, red triangles), or BFA followed by washout and recovery (BFA washout, blue squares). Horizontal lines indicate median values, bounded by interquartile ranges. *P* values, Mann Whitney *u* test. n.s, not significant.

## Supplementary Figure 5

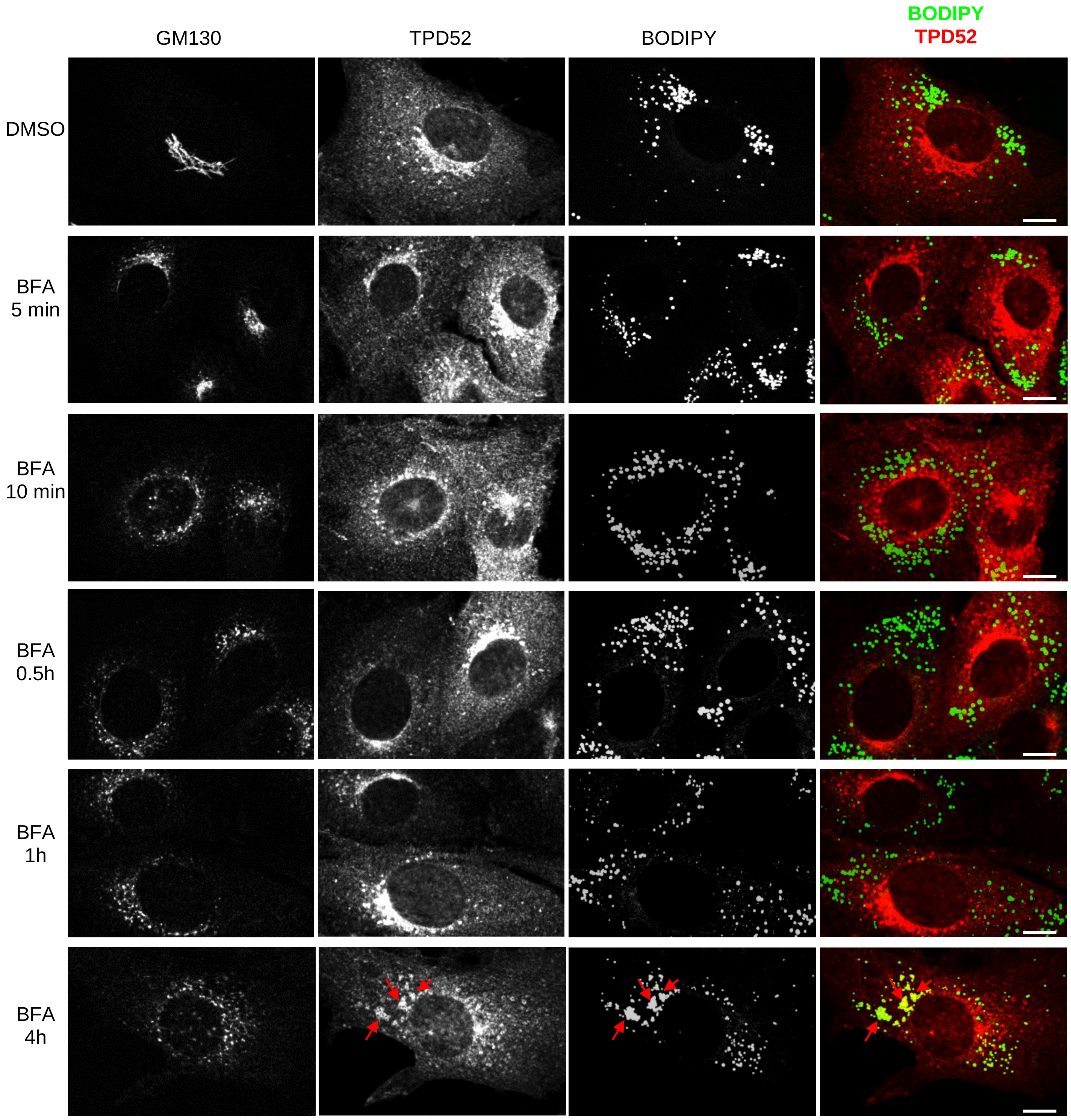


Suppl. Fig. 5. Dynamics of TPD52 recruitment to LDs in D52-2-7 cells post-BFA treatment. Immunofluorescence analyses of D52-2-7 cells treated with vehicle for 4 h (DMSO), or 2 µg/ml BFA for the indicated time periods, co-stained with BODIPY, GM130 and TPD52. Merged images of TPD52 (red) and BODIPY (green) shown at the right. Red arrows indicate co-localisation of LDs and TPD52 after 4 h BFA treatment. Images are representative of those obtained in 3 independent experiments. Scale bar = 10 µm.

## Supplementary Figure 6

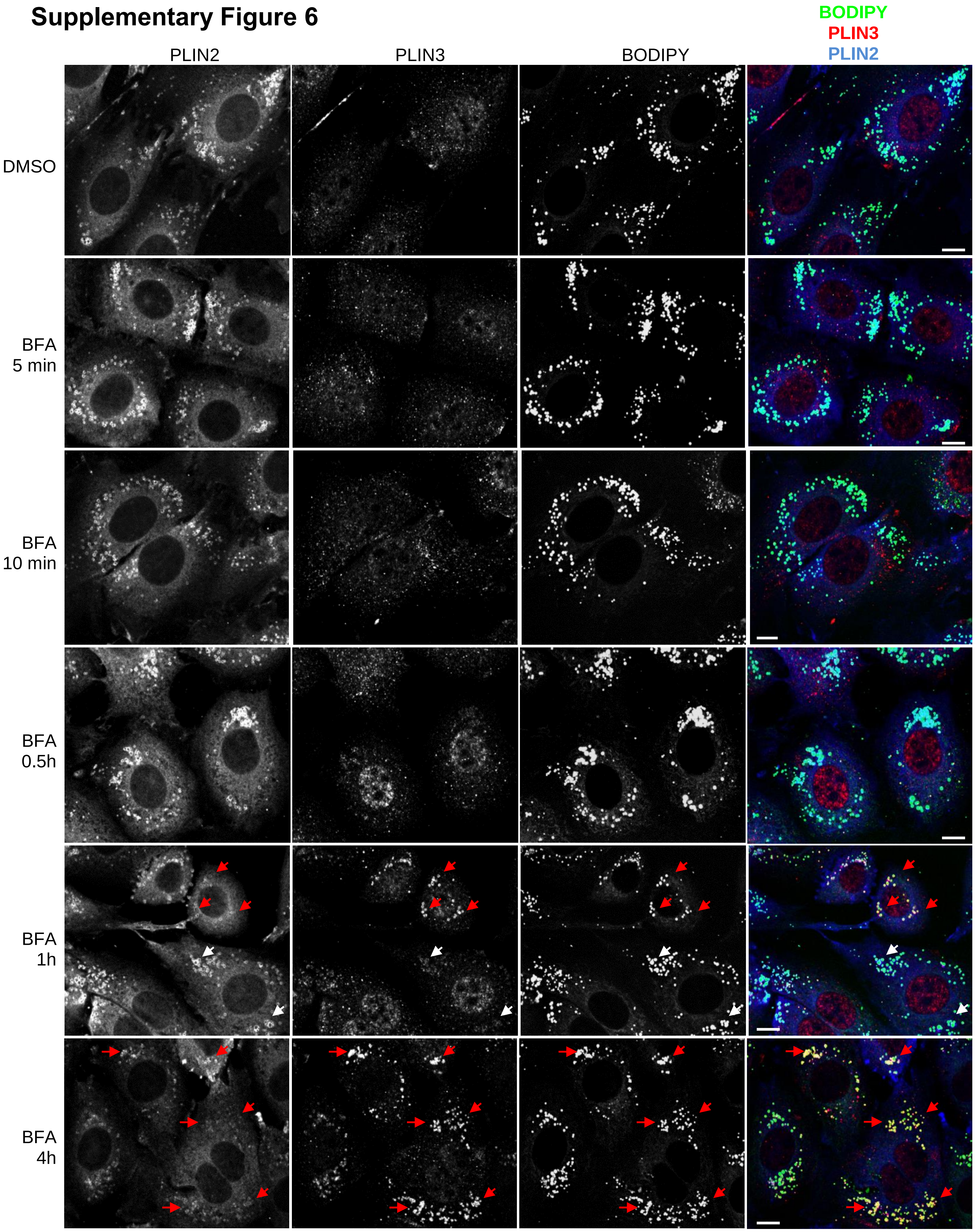


Suppl. Fig. 6. Dynamics of PLIN3 recruitment to and PLIN2 loss from LDs in D52-2-7 cells post-BFA treatment. Immunofluorescence analyses of D52-2-7 cells treated with vehicle for 4 h (DMSO), or 2 µg/ml BFA for the indicated time periods, co-stained with BODIPY (green), PLIN2 (blue) and PLIN3 (red). Merged images are shown at right. White arrows indicate LDs associated with more PLIN2 but less PLIN3 after 1 h BFA treatment, whereas red arrows indicate LDs associated with more PLIN3 but less PLIN2 after 1 h and 4 h BFA treatment. Images are representative of those obtained in 3 independent experiments. Scale bar = 10 µm.

## Supplementary Figure 7

A

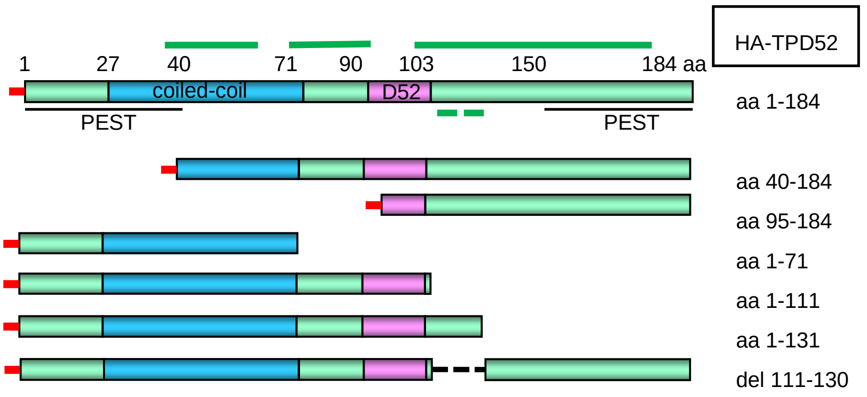


B

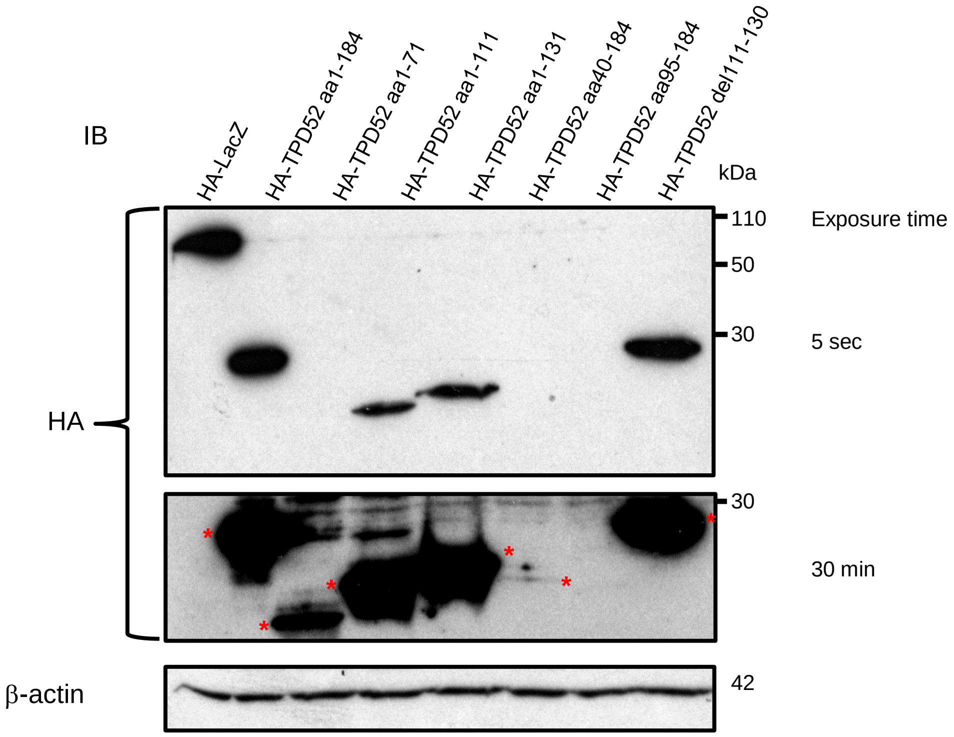


Suppl. Fig. 7. HA-TPD52 deletion constructs. (A) Schematic representations of HA-tagged TPD52 deletion mutants. Amino acid coordinates for each motif/domain are indicated. N-terminally tagged HA is indicated as a red bar. Amphipathic helices predicted by HELIQUEST (shown in Fig. 9) are indicated as green solid lines above the amino acid coordinates. The predicted ALPS-like motif (aa 111-128, shown in Fig. 9) is indicated as green dashed line below the amino acid coordinates and is deleted in HA-TPD52 del 111-130. (B) Western blot analyses of 3T3 cells transfected with pHM6 *HA-LacZ*, *HA-TPD52* full-length (aa 1-184), or *HA*-tagged *TPD52* deletion mutants. Total proteins were extracted 72 h post transfection. Transfected proteins were detected using HA antibody and indicated with red asterisks in the middle panel. β-actin served as a loading control. Molecular weights (kDa) are shown at right. Full Western blot is shown (upper panel).

## Supplementary Figure 8

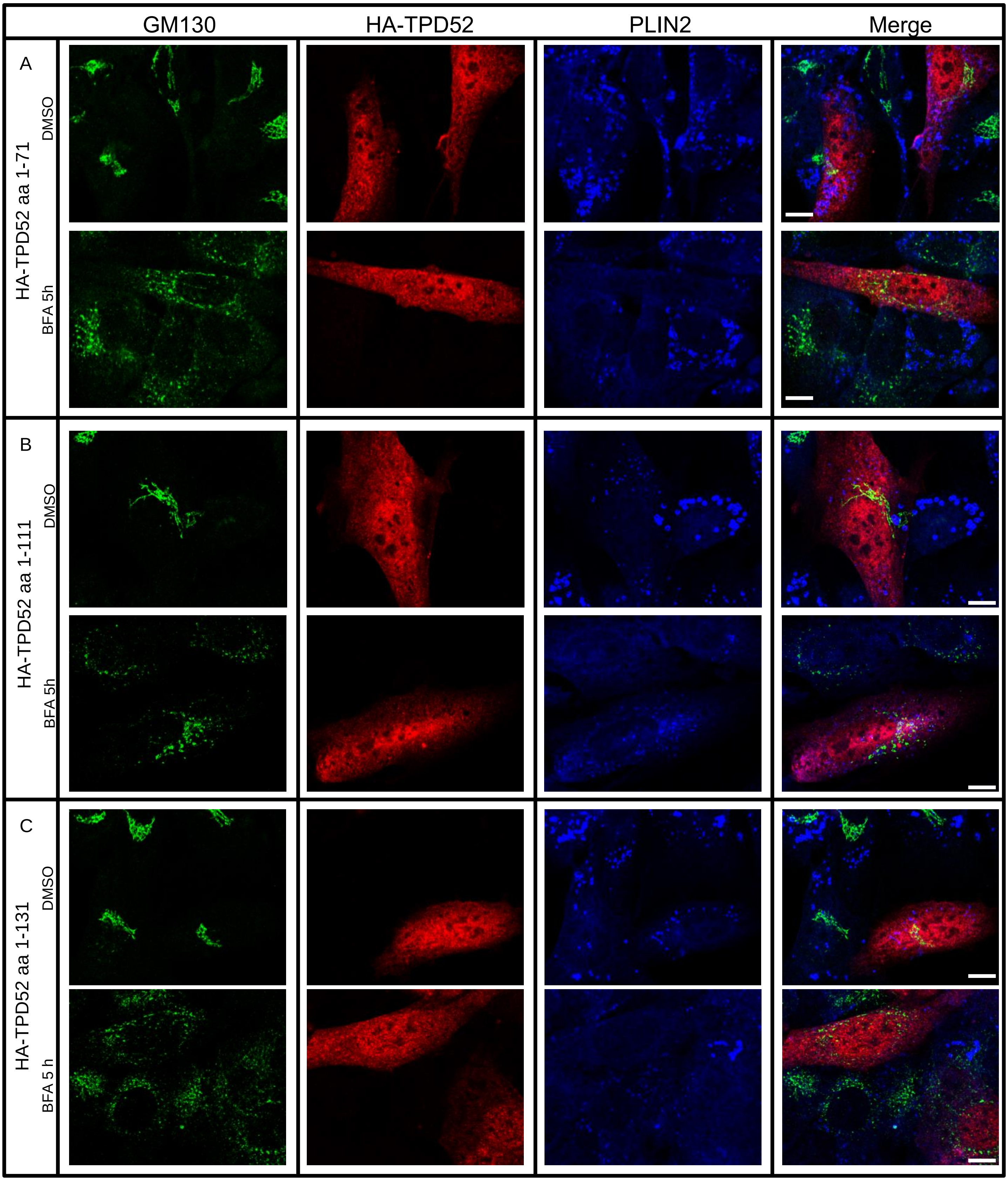


Suppl. Fig. 8. C-terminally deleted HA-TPD52 proteins were broadly detected in the cell in the presence of vehicle or BFA. Immunofluorescence analyses of 3T3 cells transfected with (A) pHM6 *HA-TPD52* aa 1-71, (B) *HA-TPD52* aa 1-111, (C) *HA-TPD52* aa 1-131 for 72 h and then treated with either vehicle (DMSO) or 2 µg/ml BFA (BFA) for 5 h, stained with GM130 (green), HA-TPD52 (red), and PLIN2 (blue). Images are representative of those obtained in 3 independent experiments. Scale bar = 10 µm.

# Supplementary Figure 9

A

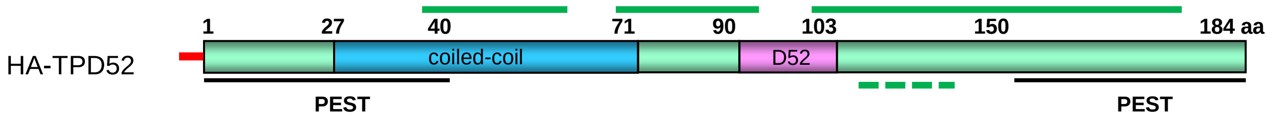


B

| Protein | Expression | DMSO | | BFA 5h | |
|---|---|---|---|---|---|
| | | Golgi | LD | Golgi | LD |
| HA-TPD52 aa 1-184 | ++++ | +++ | + | - | +++ |
| HA-TPD52 aa 1-71 | ++ | - | - | - | - |
| HA-TPD52 aa 1-111 | +++ | - | - | - | - |
| HA-TPD52 aa 1-131 | +++ | - | - | - | - |
| HA-TPD52 aa 40-184 | + | + | +++ | - | +++ |
| HA-TPD52 aa 95-184 | +/- | ND | ND | ND | ND |
| HA-TPD52 del 111-130 | ++++ | - | - | - | - |

Suppl. Fig. 9. Summary of expression levels and subcellular localisations of exogenously expressed full-length HA-TPD52 and deletion mutants. (A) Schematic representations of HA-tagged TPD52 protein as described in Suppl. Fig. 7A. (B) Summary of protein expression levels and cellular localisations of full-length HA-TPD52 and deletion mutants expressed in 3T3 cells as shown in Fig. 10 and Suppl. Figs. 7B & 8. ND, not done.

# Supplementary Figure 10

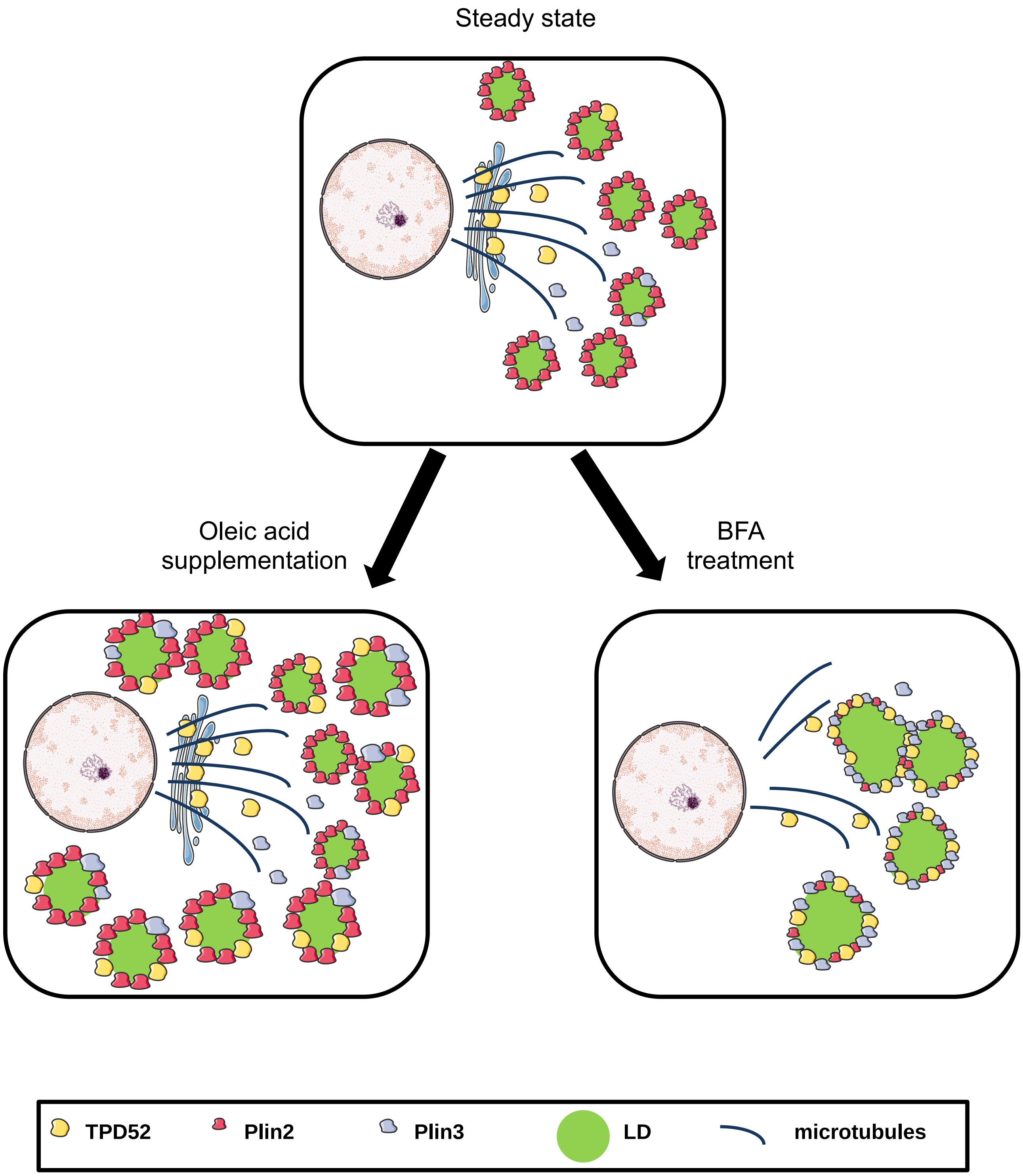


Suppl. Fig. 10. Illustration of the associations of cellular LDs with Pli2, Plin3, and TPD52 under different conditions. At steady-state, LDs are mostly coated with Plin2, whereas Plin3 and TPD52 are mainly distributed in cytoplasm with TPD52 also associated with Golgi. Upon oleic acid supplementation, the numbers and sizes of LD are increased, with more Plin2/Plin3 as well as TPD52 associated with LDs. On the other hand, BFA treatment increases LD sizes but reduces LD numbers. BFA treatment reduces Plin2 but increases Plin3 and TPD52 association with LDs. The recruitment of TPD52 onto LDs may be microtubule-dependent.

# Supplementary Figure 11

Figure 2A

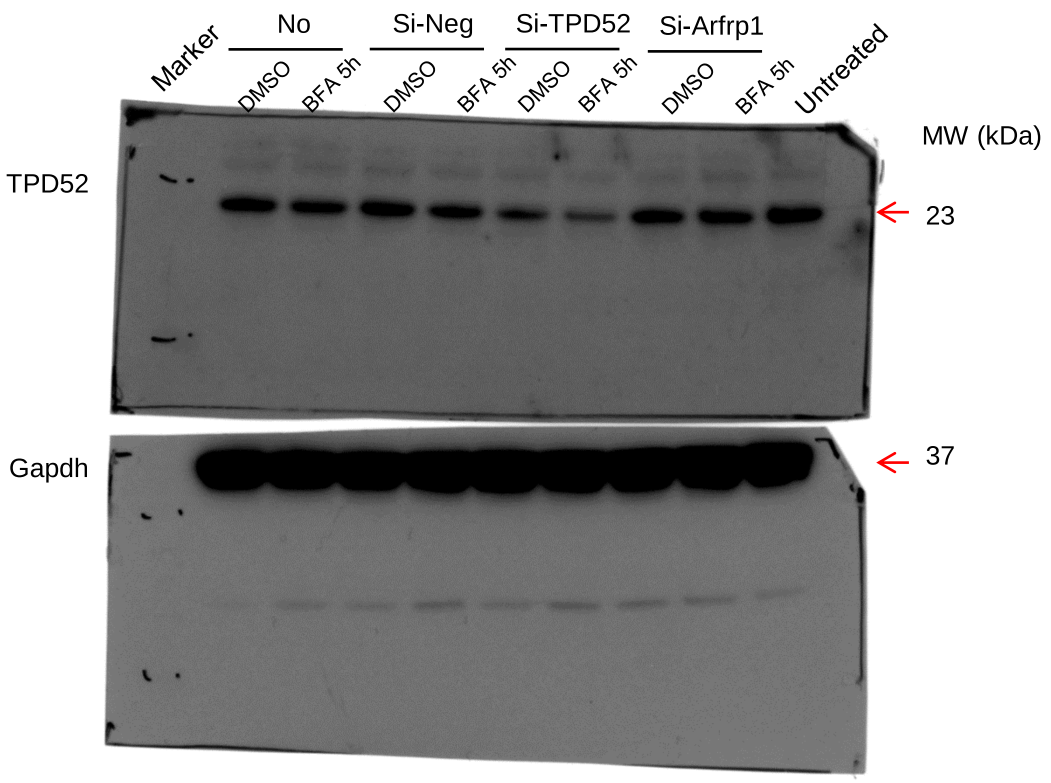


Figure 6D

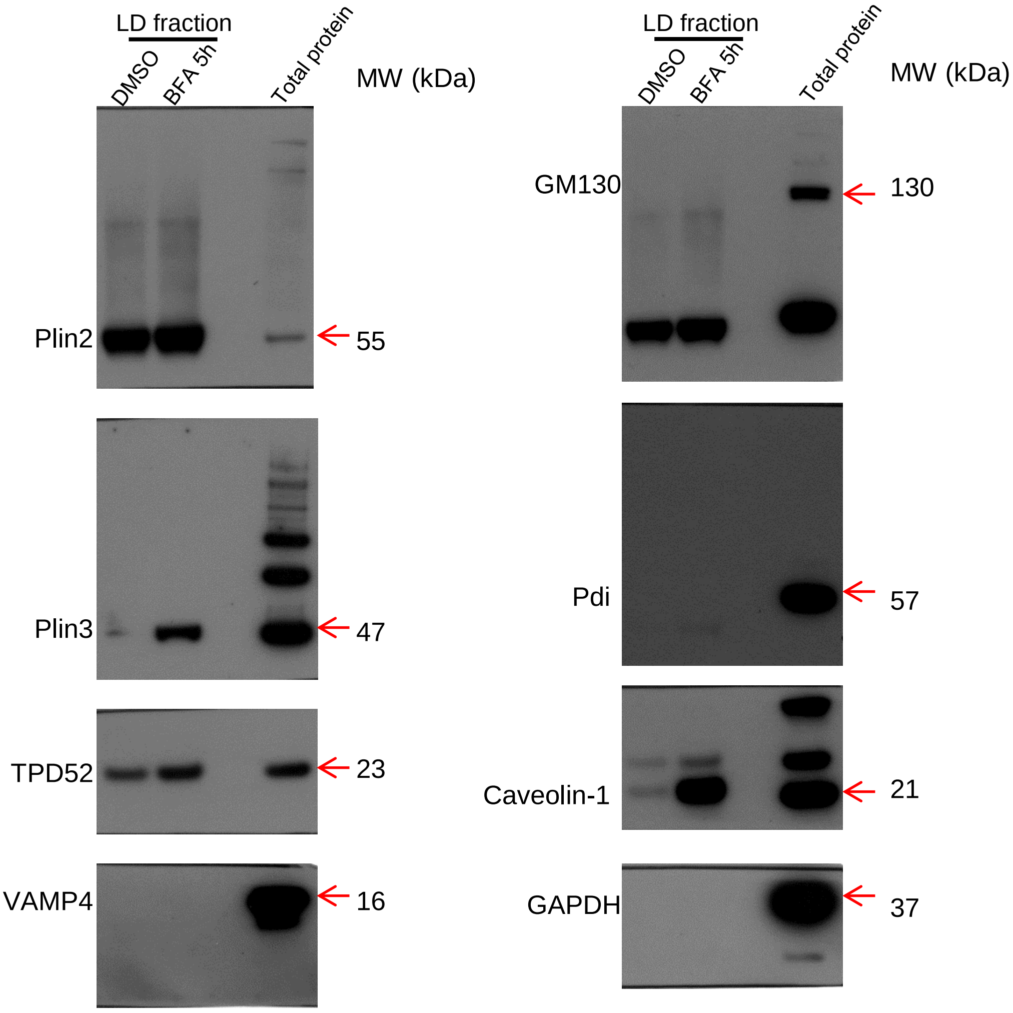

Figure 6E

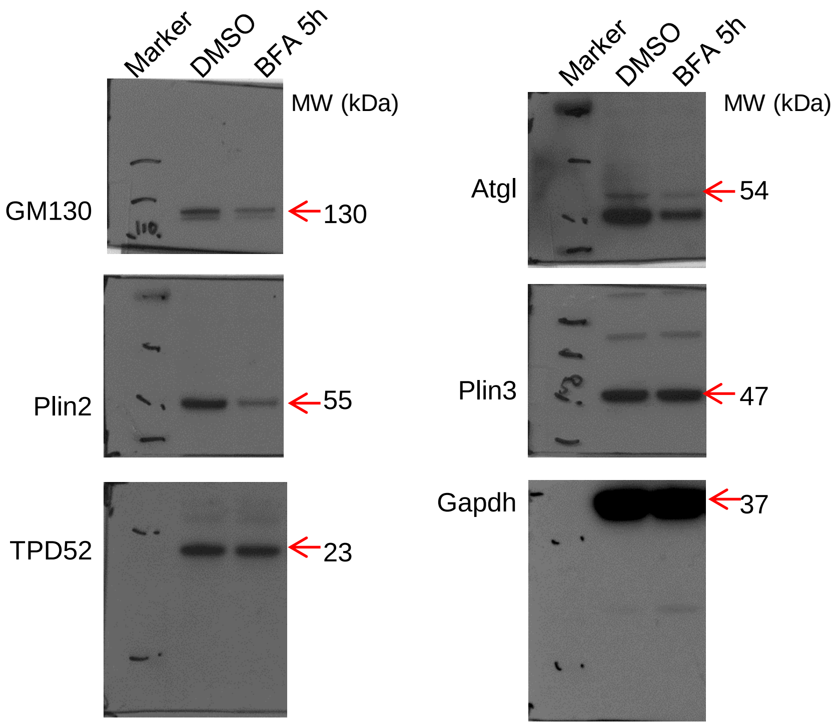


Supplementary Figure 3E

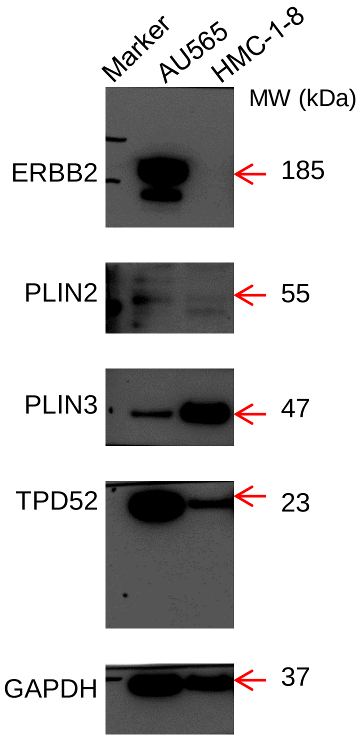


Suppl. Fig. 11. Unprocessed Western blots. Uncropped/unprocessed Western blots for Figures 2A, 6D, 6E, and Supplementary Figure 3E are shown. Note that some membranes were cut into sections to enable probing of the same blots with multiple antibodies.
